# Differential LTR-retrotransposon dynamics across polyploidization, speciation, domestication, and improvement of cotton (*Gossypium*)

**DOI:** 10.1186/s13059-025-03837-7

**Published:** 2025-10-27

**Authors:** Lucía Campos-Dominguez, Raúl Castanera, Corrinne E. Grover, Jonathan F. Wendel, Josep M. Casacuberta

**Affiliations:** 1 Centre for Research in Agricultural Genomics, CRAG (CSIC-IRTA-UAB-UB), Campus UAB, Cerdanyola del Vallès, Barcelona, Spain; 2 IRTA, Genomics and Biotechnology, Edifici CRAG, Campus UAB, Bellaterra, Catalonia 08193 Spain; 3 Department of Ecology, Evolution, and Organismal Biology, Iowa State University, Ames, IA USA

**Keywords:** Retrotransposon, Solo-LTR, Allopolyploid plants, Pangenome, TIP (transposon insertion polymorphism), Evolutionary genomics

## Abstract

**Background:**

Transposable elements are major components of plant genomes and major drivers of plant genome evolution. The cotton genus (*Gossypium*) is an excellent evolutionary model for polyploidization, speciation, domestication, and crop improvement. Here, we implement genome and pangenome analyses to study in detail the dynamics of LTR-retrotransposons during the cotton evolution.

**Results:**

We show that some LTR-retrotransposon lineages amplified in tetraploid cotton compared to their diploid progenitors, whereas others stayed stable or amplified but were removed through solo-LTR formation*.* Using species-level pangenomes we show that only a few lineages (CRM, Tekay, Ivana, and Tork) remained active after polyploidization and are still transposing. Tekay and CRM elements have re-shaped the centromeric and pericentromeric regions of tetraploid cottons in a subgenome specific manner, through new insertions but also selective eliminations through solo-LTR formation. On the other hand, Ivana and Tork have actively inserted within or close to genes affecting their expression. Finally, population-level analyses using the two pangenomes and data from 283 and 223 varieties of *G. hirsutum* and *G. barbadense* reveal changes in Transposon Insertion Polymorphism frequencies accompanying domestication and improvement of both species, suggesting the possibility of selection on linked regions.

**Conclusions:**

Our findings reveal that LTR-retrotransposon lineages followed differential dynamics during cotton evolution, displaying differences among species and the two coresident genomes of allopolyploid cotton. A handful of the LTR-retrotransposon lineages that expanded after polyploidization helped shape the genomes of both *G. hirsutum* and *G. barbadense*, impacting their centromere and pericentromeric regions as well as protein-coding genes.

**Supplementary Information:**

The online version contains supplementary material available at 10.1186/s13059-025-03837-7.

## Background

Transposable elements (TEs) are major components of plant genomes. Their repetitive nature and their ability to amplify and move within the genome make them a major source of genetic variability and one of the major drivers of plant genome evolution [1, 2]. TEs are an important source of mutations underlying crop domestication and improvement [3]. However, as potential mutagens, TEs are tightly controlled, mainly through epigenetic mechanisms [4]. For this reason, the dynamics of TEs within genomes have often been seen as an arms race between an invasive parasite and a defensive host, with genomes tightly controlling TE activity and TEs evolving to evade genomic suppression [5–7]. TE activity is known to increase in situations where DNA methylation and epigenetic silencing decrease, such as stress situations [8], including the genomic shock that results from the combination of two different genomes in polyploid species [9, 10]. This activation can lead to a burst of transposition and a subsequent repression when silencing is re-established [11]. Yet accumulated data during the last few years in plant genomes suggests that the dynamics of different TE types, lineages, and families that coexist in a genome are highly diverse. These lineages and families may thus be viewed as different players in an evolving ecosystem [12–14].

Long terminal repeat retrotransposons (LTR-retrotransposons) are the most prevalent type of TEs in plants, as exemplified by maize, where LTR-retrotransposons account for 90% of the TEs and more than 80% of the genome space [14]. LTR-retrotransposons are divided into two major superfamilies, *Copia* and *Gypsy*, which often show important differences in their regulation and distribution within genomes [15]. Within these two superfamilies, LTR-retrotransposons can be further subdivided into different lineages [16]. Elements of the same lineage may have similar functional characteristics. As an example, CRM elements are frequently associated with the centromeres of different plant species [17]. However, elements of the same lineage can also show important differences in different host genomes [12].

LTR-retrotransposons transpose through a replicative mechanism, i.e., via an RNA copy reverse transcribed into cDNA prior to integration at a new location. Therefore, their activity results in an increase in copy number, which endows them with the potential to invade, and thereby inflate, the genome. As an example, the genome of the wild rice *O. australiensis* doubled in size in just three million years by the amplification of only three families of LTR-retrotransposons [18]. Counterbalancing this genome size expansion via TE proliferation, LTR-retrotransposons can also be eliminated from genomes by different mechanisms [19, 20]. The main mechanisms for this deletional process are illegitimate recombination, resulting in truncated LTR-retrotransposon copies, and unequal homologous recombination at the LTRs, giving rise to so-called solo-LTRs [21–25]. Although the importance of recombination-mediated LTR-retrotransposon elimination is well recognized, its analysis is often neglected when studying LTR-retrotransposon dynamics.

The cotton genus (*Gossypium*) serves as an excellent evolutionary model for polyploidization, domestication, and crop improvement. Allopolyploid cotton species arose 1–2 mya from the interspecific hybridization of an “A-genome” and a “D-genome” species, which diverged from each other 5–7 mya [26]. Because of this long divergence of the two genome donors, A and D, their resulting subgenomes can be easily distinguished in the tetraploid genomes. The ancestor providing the A subgenome is closely related to the modern species *G*. *herbaceum* (A1) and *G. arboreum* (A2), whereas the ancestor of the D subgenome is closest, among extant D-genome species, to *G. raimondii* (D5) [26]. Two allotetraploid species (*G. hirsutum*, AD1, and *G. barbadense*, AD2) were independently domesticated from different wild progenitors in Central and South America, respectively, over the last 8000 years [26, 27].

TE studies on cotton species have previously highlighted their significant role in genome size expansion, being the major contributors to genome size differences between diploid cotton species [28]. Recent analyses have also suggested that TEs may have amplified accompanying the polyploidization event, particularly for the *Gypsy* Tekay and CRM lineages of LTR-retrotransposons [29], which may have reshaped the centromeres in tetraploids [30].

Here we study the LTR-retrotransposon dynamics (both LTR-retrotransposon insertion and removal through solo-LTR formation) accompanying diploid divergence, polyploidization, and speciation at the allopolyploid level using genome assemblies of eight *G. hirsutum* and ten *G. barbadense* accessions and representatives of their parental diploid species. Using pangenomes generated for the two domesticated tetraploid cotton species (*G. hirsutum* and *G. barbadense*), we report on recent LTR-retrotransposon dynamics accompanying initial crop domestication and subsequent improvement. Using this temporally stratified and explicit approach, we show that each LTR-retrotransposon lineage has experienced its own dynamics throughout the different events characterizing the recent evolution of cotton species and varieties. We also use the pangenomes to analyze relatively recent or ongoing transposition events within the two allopolyploid species, documenting the frequency of the intraspecies transposon insertion polymorphisms (TIPs) in approximately 500 sequenced genomes representing the wild-to-domesticated continuum within *G. hirsutum* and *G. barbadense.* Our results show that LTR-retrotransposons may have been instrumental in shaping cotton genomes and generating the genome variability that was subject to selection during domestication and crop improvement.

## Results

### LTR-retrotransposon lineages have differentially amplified in tetraploid cotton species

As a first step in exploring the dynamics of LTR-retrotransposons in the recent evolution of cotton, we annotated intact LTR-retrotransposon copies in the highly contiguous assemblies of eight accessions of *G. hirsutum* and ten accessions of *G. barbadense*, as well as in the genomes of two diploid species that represent the best models of the ancestral A (*G. herbaceum* and *G. arboreum*) and D subgenomes (*G. raimondii*) of cotton. Our analysis focused only on the intact LTR-retrotransposon copies (identified by stringent approaches; see the “ Methods” section) to concentrate on the youngest elements, i.e., those that could have arisen from transposition accompanying polyploidization, speciation, and domestication of cotton, events that occurred within the last 1–2 million years.

**Fig. 1:**
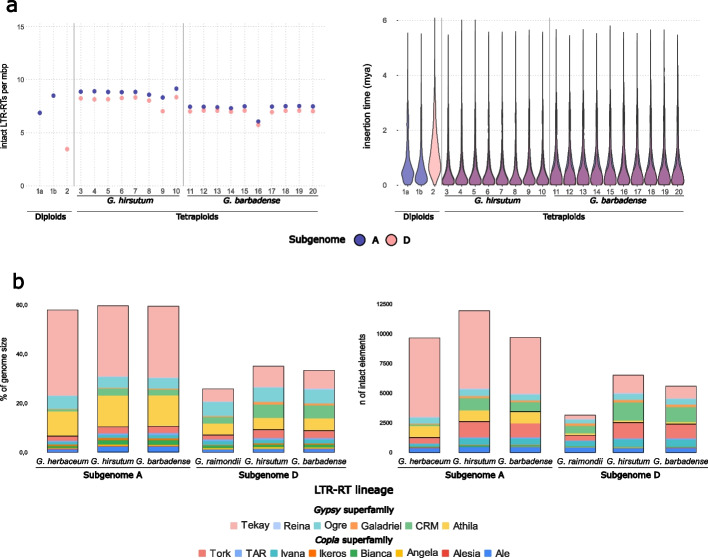
LTR-retrotransposon content in diploid and tetraploid cottons by subgenome. **a** Number of intact LTR-retrotransposons per megabase pairs per subgenome in each of the cotton species and varieties analyzed (left panel), and plots representing the insertion times intact LTR-retrotransposons per subgenome in each of the cotton species and varieties analyzed (right panel). Numbers on the *X*-axis correspond to genome assemblies as listed here: (1a) G. herbaceum A1a (wild); (1b) G. arboreum SYX1; (2) G. raimondii GPD5lz; (3–10) G. hirsutum accessions: TX-1000 (3), TX2094 (4), B713 (5), Bar32 (6), NDM8 (7), TM1 (CRI version) (8), ZM24 (9), CRI12 (10); (11–20) G. barbadense accessions: GB0333 (11), GB0414 (12), GB0660 (13), GB0776 (14), K101 (15), 3–79 (16), Pima90 (17), Giza7 (18), Junhai-1 (19), and Yuma (20). Assembly information is detailed in Additional file 2: Table S1. **b** Lineage classification of the intact LTR-retrotransposon elements of each species and subgenome. The values of the tetraploid *G. hirsutum* and *G. barbadense* species are represented by the average values of all varieties of the corresponding species analyzed (left panel). Total assembled genome % corresponding to each LTR-retrotransposon lineage in the diploid and tetraploid cotton species analyzed. The tetraploid *G. hirsutum* and *G. barbadense* genomes are represented by the varieties TM1 and Pima90, respectively (right panel)

Results in Fig. 1a (left panel) show that the two polyploid species have, in general, a higher number of intact LTR-retrotransposons per megabase than do the diploids, especially in the D subgenome, where the density of intact LTR-retrotransposon elements is roughly doubled (from 3.5 elements per Mbp in the D-genome diploid parental *G. raimondii* to ~ 8 and ~ 7 elements per Mbp in *G. hirsutum* and *G. barbadense*, respectively). For the A subgenome, there is an increased density with respect to that of *G. herbaceum* but not with respect to *G. arboreum*. However, it has been shown that *G. arboreum* experienced a recent increase in LTR-retrotransposon copy number, and therefore its present LTR-retrotransposon density does not reflect its density when polyploidization occurred [31]. Therefore, we only use *G. herbaceum* as a comparator for the A subgenomes henceforth. Between the two tetraploid species, *G. hirsutum* varieties generally have a slightly higher number of intact LTR-retrotransposons than the *G. barbadense* accessions, and there are no major differences among accessions within each of the two species (Fig. 1a, left panel).

Insertion times were estimated for each element by comparing the two LTR sequences for each intact LTR-retrotransposon. Figure 1a (right panel) shows that LTR-retrotransposon insertions are younger, on average, in the polyploids than in the diploids, suggesting more recent activation of LTR-retrotransposons in the polyploids. Analysis of the LTR-retrotransposon composition of the genome of the two tetraploid species relative to their two diploid parentals reveals that the increase in intact copies, and therefore in the percentage of the genome covered by LTR-retrotransposons, is mainly due to a few LTR-retrotransposon lineages (e.g., CRM and Tork, see Fig. 1b).

**Fig. 2:**
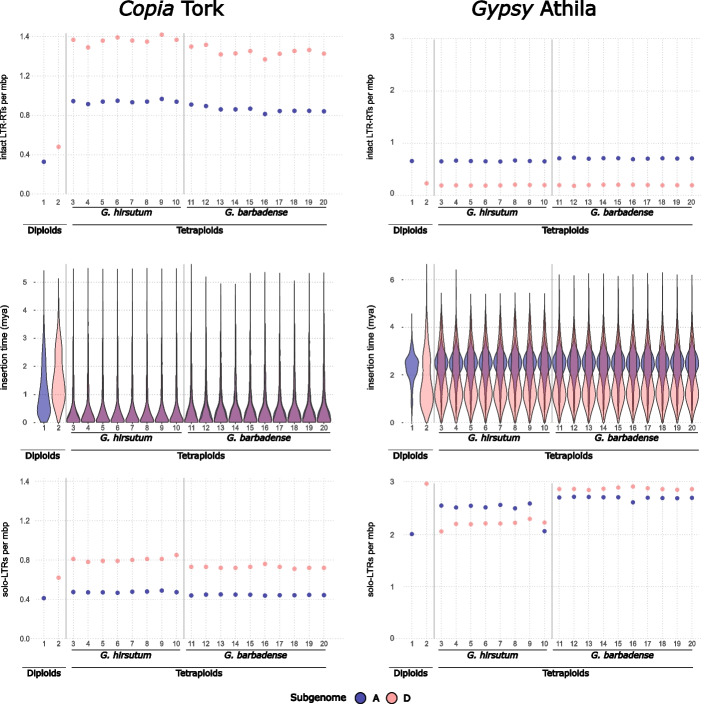
Intact LTR-retrotransposons and solo LTR *Copia* Tork and *Gypsy* Athila content in diploid and tetraploid cottons by subgenome. Number of intact LTR-retrotransposons per megabase pairs per subgenome in each of the cotton species and varieties analyzed (top panels), violin plots representing the insertion times these intact LTR-RTs per subgenome in each of the cotton species and varieties analyzed (middle panels), and number of solo LTRs per megabase pairs per subgenome in each of the cotton species and varieties analyzed. (bottom panels). *Y*-axis scales differ between subpanels due to the large variation in abundance among LTR-retrotransposon lineages. This allows for better visualization of less abundant lineages. Within each subpanel, intact elements and solo LTRs are shown on the same scale to enable direct comparison. Numbers on the *X*-axis correspond to genome assemblies as in Fig. 1a

**Fig. 3:**
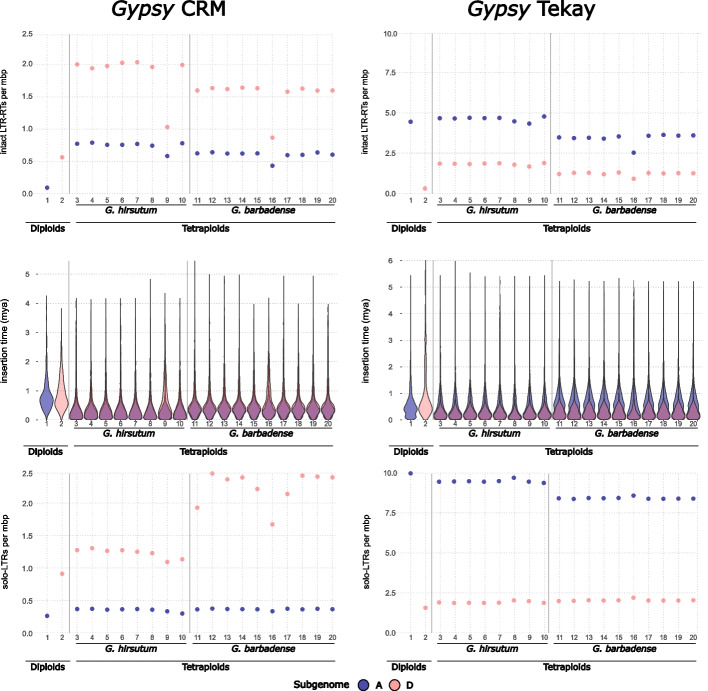
Intact LTR-retrotransposons and solo LTR *Gypsy* CRM and *Gypsy* Athila content in diploid and tetraploid cottons by subgenome. Number of intact LTR-RT per megabase pairs per subgenome in each of the cotton species and varieties analyzed (top panels), violin plots representing the insertion times these intact LTR-RTs per subgenome in each of the cotton species and varieties analyzed (middle panels), and number of solo LTRs per megabase pairs per subgenome in each of the cotton species and varieties analyzed (bottom panels). *Y*-axis scales differ between subpanels due to the large variation in abundance among LTR-retrotransposon lineages. This allows for better visualization of less abundant lineages. Within each subpanel, intact elements and solo LTRs are shown on the same scale to enable direct comparison. Numbers on the *X*-axis correspond to genome assemblies as in Fig. 1a

Analysis of the number of intact elements from each LTR-retrotransposon lineage (Figs. 2 and 3 and Additional file 1: Fig. S1) indicates that there is diversity in dynamics even among lineages belonging to the same superfamily of LTR-retrotransposons (i.e., *Copia* and *Gypsy*), whereby some lineages exhibit abundant increases in copy number in the polyploids, whereas others remain relatively stable during recent cotton evolution. For example, the copy number for the *Copia* Tork lineage is three times higher per megabase in the two tetraploids than in the two diploid progenitors (Fig. 2 top, left panel). Insertion time analysis of Tork elements shows that although most elements are relatively old in the diploids, they are notably younger in the polyploids (Fig. 2 middle, left panel). This confirms that Tork has been particularly active in the tetraploid genomes and may still be actively transposing. In contrast, the number of copies per megabase of other lineages, such as the relatively abundant *Gypsy* Athila (Fig. 2 top, right panel), is similar when comparing each subgenome of the polyploid with its corresponding diploid genome, suggesting no or little active retrotransposition since polyploid formation. An analysis of the insertion time of Athila intact elements shows a relatively old insertion time, particularly for elements residing in the A subgenomes, and an almost identical distribution of insertion times between diploid and tetraploid genomes, supporting very low activity after polyploidization (Fig. 2 middle, right panel). However, Athila elements have probably been very active in the past, as Athila-related sequences account for a sizeable fraction (~ 13% and 5% in subgenomes A and D, respectively) of the genome of both tetraploid species and their diploid parentals (~ 10% in *G. herbaceum* and 4.6% in *G. raimondii*) (Fig. 1b).

A general inference from the analysis of copy number for all LTR-retrotransposon lineages is that the evolutionary behavior of the different TE families varies considerably among lineages, subgenomes, species, and with respect to the timing of proliferation events (Figs. 2 and 3 and Additional file 1: Fig. S1). Some LTR-retrotransposon lineages show an increase in retrotransposition in both subgenomes after polyploidization (the *Copia* Tork and Ivana, and the *Gypsy* CRM), whereas others mainly increased in one subgenome (the *Gypsy* Tekay). In contrast, a stable number of LTR-retrotransposon insertions was observed among all species at both the diploid and tetraploid levels for some retrotransposon lineages, such as the *Gypsy* Athila, Ogre, Reina, Galadriel families, and the *Copia* Ale family. Finally, some TE families are present at similarly low copy numbers in all genomes (the *Copia* Bianca, Ikeros, and TAR). In general, lineages present at a relatively high copy number exhibited similar insertion numbers when comparing different varieties of the same species, although this is not always the case, as evidenced by the Ivana lineage in the subgenome D of *G. hirsutum* (Additional file 1: Fig. S1)*.* Similarly, the copy number of each lineage is generally similar between the two tetraploid species, although differences for specific lineages do exist. Notably among these exceptions are the CRM and Tekay *Gypsy* lineages (Fig. 3), which are further discussed below.

### The rate of LTR-retrotransposon elimination varies among lineages, species and subgenomes

We further explored the dynamics of LTR-retrotransposon elements accompanying recent cotton evolution by estimating LTR-retrotransposon elimination through intra-element LTR-recombination. We annotated potential solo-LTRs by searching for LTRs corresponding to the lineages and families of intact LTR-retrotransposons that did not have surrounding sequences that could correspond to LTR-retrotransposon internal sequences (see the “ Methods” section). We note, however, that truncated LTR-retrotransposon elements with small regions of insufficient similarity to LTR-retrotransposon internal regions will also be included in the solo-LTR annotation. The approach followed does not allow for a precise annotation of the solo-LTR borders that would allow looking for the possible presence of Target Site Duplications (TSDs), a hallmark of recent solo-LTRs. However, the pangenome analysis of *G. hirsutum* and *G. barbadense* described in a subsequent section allowed for a precise analysis of polymorphic solo-LTRs and showed that 95% and 91% of them, respectively, in these two pangenomes were flanked by TSDs (see below), suggesting that a major fraction of the putative solo-LTRs here described are genuine solo-LTRs.

The analysis of the annotated solo-LTRs shows that there are important differences among the LTR-retrotransposon lineages (Figs. 2 and 3 and Additional file 1: Fig. S1, bottom panels). For example, the relative number of Tork solo-LTRs is similar between tetraploid and diploid genomes (Fig. 2 bottom, left panel), despite the increase in intact Tork insertions in polyploid cotton relative to their parental diploids (Fig. 2 top, left panel). Combined with the younger age of intact Tork elements in the tetraploids (relative to the diploids; Fig. 2 middle, left panel), these observations suggest both that Tork elements have been more active in the tetraploids than in the diploids (after polyploidization) and that only a minor fraction of the new insertions has been removed by recombination. In contrast, both diploids and tetraploids contain two to ten times more Athila solo-LTRs than intact elements (Fig. 2 bottom, right panel), suggesting a high rate of elimination of these elements. As noted above, Athila has similarly old element insertion times between diploids and polyploid genomes (Fig. 2 middle, right panel), suggesting low or no recent activity of this family; however, the greater number of solo-LTRs in *G. barbadense* relative to *G. hirsutum* may indicate recent activity and rapid turnover of Athila insertions in *G. barbadense*.

A similar diversity of solo-LTR dynamics characterizes the remaining LTR-retrotransposon lineages. For example, Ogre solo-LTRs are also more abundant than intact elements (Additional file 1: Fig. S1); however, unlike Athila, intact elements tend to be younger in the tetraploids, particularly for those in the D subgenome of *G. hirsutum*, where the number of solo-LTRs is particularly high. This observation may suggest that the removal of intact elements through solo-LTR formation affected mostly older elements. On the other hand, Ivana, very much like Tork, shows a low number of solo-LTRs and a high number of young intact elements, suggesting active retrotransposition and low recombination rates. In contrast, Angela, Sire, TAR, Bianca, and Ikeros families have more solo-LTRs than intact elements, although the numbers are all low, suggesting limited retrotransposition activity for each of these families. Finally, Ale, Galadriel, and Reina show the same number of intact LTR-retrotransposons and solo-LTRs in the tetraploids as in the parental diploids, and similar LTR-retrotransposon age, suggesting little or no activity during the recent evolution of *Gossypium*.

### CRM and Tekay insertion and elimination have shaped the centromeres of tetraploid cottons

The CRM and Tekay lineages have previously been shown to have amplified in the tetraploids [29] and have been suggested to play a role in the reorganization of their centromeres [30]. Our results confirm that CRM and Tekay have been actively transposing after the polyploidization event, as deduced by their recent insertion time distribution compared to the diploids (Fig. 3), which is also supported by the phylogenetic analysis of the LTR-RT domain of these lineages found across diploids and tetraploid genome assemblies (Additional file 1: Fig. S2). Moreover, our results also show that CRM copy number increased greatly in the A subgenomes of both tetraploids (up to eightfold in *G. hirsutum* and up to 6.5-fold in *G. barbadense*) and to a lesser extent in the D subgenomes (up to 2.6-fold in *G. hirsutum* and up to twofold in *G. barbadense*), with this increase being slightly higher in *G. hirsutum* than in *G. barbadense* (Fig. 3 top, left panel). A different pattern was observed for Tekay elements, which exhibited increased copy numbers in the D subgenomes of both tetraploids (also slightly more in *G. hirsutum* than in *G. barbadense*), but not in the A subgenomes, where its copy number remained high and stable in *G. hirsutum* and decreased about 30% in *G. barbadense* (Fig. 3 top, right panel). These results thus show that, whereas both CRM and Tekay actively transposed accompanying polyploidization, their amplification rate was dissimilar and differed between species and subgenomes.

Analysis of the CRM and Tekay solo-LTRs shows that the elimination rate also varies between the two polyploids and between subgenomes (Fig. 3 bottom panels). The number of CRM solo-LTRs is much higher in the D subgenomes than in the A subgenomes in both polyploids, but it is particularly high in *G. barbadense*. This could be the consequence of a higher elimination rate of recent CRM insertions from the D subgenomes, particularly in *G. barbadense*, which would also explain the lower number of intact CRMs in the D subgenomes of this species (Fig. 3 top, left panel) and the slightly older insertion time of the remaining *G. barbadense* CRMs (Fig. 3 middle panels). Interestingly, the analysis of the Tekay lineage suggests an opposite dynamic. Tekay solo-LTRs are much more abundant in the A subgenomes of both tetraploids than in the D subgenomes, a trend that is even more extreme for *G. hirsutum* (Fig. 3 bottom, right panel). Interestingly, the high number of Tekay solo-LTRs, doubling that of complete elements, is also seen in the diploid parental, *G. herbaceum*, which may suggest a link between the nature of the A subgenomes and the efficiency of Tekay recombination. This high elimination rate of Tekay elements in the A subgenomes could explain why the recent activity suggested by the very recent age of the intact elements (Fig. 3 middle, right panel) has only resulted in an increase of Tekay insertion in the D subgenomes (Fig. 3 top, right panel).

**Fig. 4:**
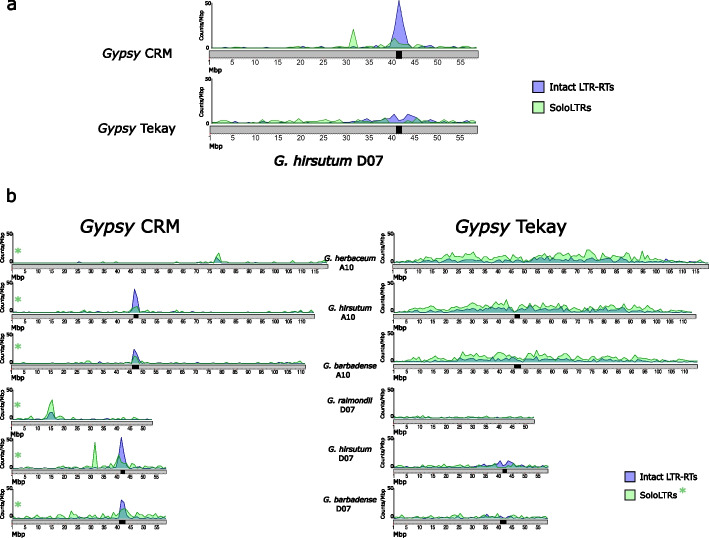
Chromosomal distribution of CRM and Tekay elements. **a** Density plots (number of elements per 1Mbp sliding window) of CRM and Tekay intact LTR-retrotransposons and solo-LTRs along chromosome D07 of *G. hirsutum*. **b** Density plots (number of elements per 1Mbp sliding window) of CRM and Tekay intact LTR-retrotransposons and solo-LTRs along chromosomes A10 and D07 in the diploid and tetraploid cottons here analysed. A green asterisk indicates where the scale of the *Y*-axis of solo-LTRs represented has been modified (2 ×) in order allow visualizing their distribution along chromosomes

The distribution of the intact CRM elements and solo-LTR CRM shows a high concentration of CRM intact copies in the regions characterized as containing the centromeres across all chromosomes [30], whereas CRM solo-LTRs are more homogeneously distributed along the chromosomes (Fig. 4a, Additional file 1: Fig. S3), suggesting that, although CRM elements may target the centromere [32], their differential elimination from other chromosome regions that do not show the recombination suppression typical of centromeres [32] reinforces their concentration in the centromeric regions. Similarly, intact Tekay elements in the D subgenomes are concentrated in the pericentromeric regions, whereas Tekay solo-LTRs are not, suggesting active elimination of Tekay elements from chromosomal regions maintains their prevalence in pericentromeric regions. Interestingly, Tekay elements are not found in CRM-rich regions of the centromere, perhaps reflecting an insertional preference difference between these two types of elements. The distribution of Tekay intact and solo-LTRs in the A subgenomes seems less skewed than in the D subgenomes, although they also show an opposite distribution with respect to genes.

Comparison of the CRM and Tekay distributions between the diploids and the tetraploids also reveals notable differences. First, the high increase in CRM copy number in the A subgenomes of the two polyploids, and particularly in *G. hirsutum*, is concentrated almost exclusively in the centromeric region of every chromosome, whereas in the diploids, the distribution of the CRM elements on each chromosome is less skewed (Fig. 4b, and Additional file 1: Fig. S4). Second, the solo-LTR distribution in the diploids parallels that of the intact copies across all chromosomes, suggesting that there is no preferential elimination of CRM elements from chromosome arms (Fig. 4b and Additional file 1: Fig. S4). For Tekay elements, the increase in intact elements in the D subgenomes of polyploids (up to fivefold in *G. hirsutum* and up to 3.5-fold in *G. barbadense*) without a concomitant increase in the number of solo-LTRs appears to concentrate the intact elements in the pericentromeric regions across all chromosomes (Fig. 4b and Additional file 1: Fig. S4).

### A small number of LTR-retrotransposon lineages accounts for intraspecies polymorphisms

Although the number of insertions of most LTR-retrotransposon lineages appears relatively constant within each tetraploid species, the young age of the insertions for many LTR-retrotransposon lineages suggests recent retrotransposition. Therefore, to understand the scale and scope of TE activity within species, we constructed reference-based pangenomes for both species that we subsequently used to characterize transposon insertion polymorphisms (TIPs) within *G. hirsutum* and *G. barbadense*. We used genome assemblies of seven different varieties to build the *G. hirsutum* pangenome and genome assemblies of nine different varieties to build the *G. barbadense* pangenome (Additional file 2: Table S1). The different genome sequences were aligned to the references for each species (TM-1 and 3–79) to build each pangenome. The alignments covered 94.1–98.7% of the *G. hirsutum* TM1 reference and 94.8–97.0% of the *G. barbadense* 3–79 reference. The number of structural variants (SV) detected in the *G. hirsutum* and *G. barbadense* pangenomes was 63,081 and 55,122, respectively, most of which (57,727 and 48,990, respectively) were insertion/deletion (indel) polymorphisms. Approximately 20% of these indels (12,316 *in G. hirsutum* and 9386 in *G. barbadense*) contained LTR-retrotransposon sequences (at least 30% coverage of an LTR-retrotransposon). To detect recent LTR-retrotransposon insertions, we defined LTR-retrotransposon TIPs as indel SVs with at least 80% identity over 80% of the length of an intact LTR-retrotransposon representative or a solo-LTR, and a minimum size of 80% of an intact LTR-retrotransposon or a solo-LTR. This analysis led to the detection of 4436 LTR-retrotransposon TIPs in *G. hirsutum* and 3520 in *G. barbadense*, and 2406 solo-LTR TIPs in *G. hirsutum* and 1313 in *G. barbadense*. An analysis of the reference genomes of each species (TM-1 and 3–79) showed that 95% of *G. hirsutum* and 94% of *G. barbadense* LTR-retrotransposon TIPs and 95% of the *G. hirsutum* and 91% of *G. barbadense* solo-LTR TIPs have TSDs, confirming that they are the result of retrotransposition (followed by unequal homologous recombination in the case of solo-LTR TIPs). Both the SVs in general and the TIPs (from LTR-retrotransposons and solo-LTR) were homogeneously distributed along chromosomes (Additional file 1: Fig. S5a).

**Fig. 5:**
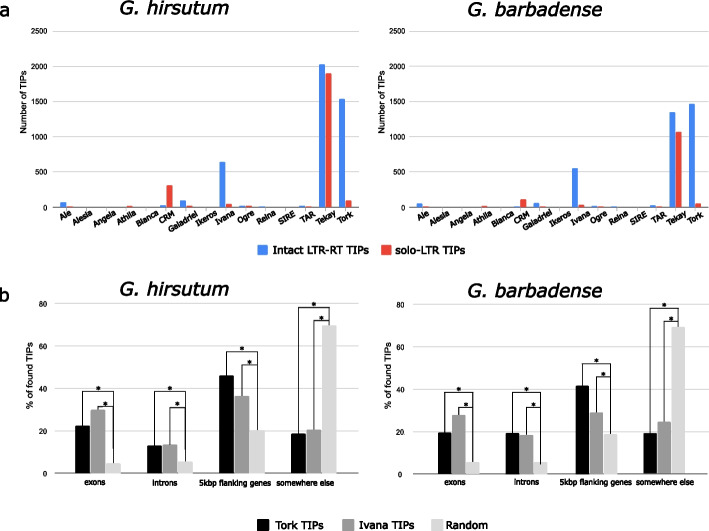
Intraspecific variability of LTR-retrotransposons and solo-LTR insertions. **a** Number of TIPs corresponding to intact LTR-retrotransposon and solo-LTR insertions in the *G. hirsutum* and *G. barbadense* pangenomes per LTR-retrotransposon lineage. **b** Percentage of Tork and Ivana TIPs in different genome regions as compared with a distribution at random (percentage of the genome occupied by each region). Asterisks represent significant enrichment/depletion of insertions in each region compared to random insertions (Chi2 test, *p*-value < 0.05)

In addition to this overall quantitative view of TE-mediated TIPS, LTR-retrotransposon lineage classification of both intact and solo-LTR TIPs shows that only a few of the many LTR-retrotransposon lineages are responsible for the polymorphism generated within the two cotton species (Fig. 5a and b, Additional file 2: Tables S3, S4). Interestingly, some lineages that experienced copy number increase in tetraploid cotton relative to their diploid progenitors, e.g., Ogre (Additional file 1: Fig. S1), also show little variability within the polyploids (less than 1%, Additional file 2: Tables S3, S4 and Fig. 5a), suggesting that they experienced a burst of transposition following polyploidization but became silent before the split of the two tetraploids from their common ancestor. In contrast, some lineages appear to have retained high transpositional activity after the speciation of the two polyploids. These include the *Gypsy* CRM and Tekay lineages and the *Copia* Ivana and Tork lineages (Fig. 5a). Interestingly, CRM is mainly polymorphic as solo-LTRs, whereas Ivana and Tork are mainly polymorphic as complete LTR-retrotransposons and Tekay is notably polymorphic as both. These patterns could suggest recent elimination activity for CRM, recent retrotransposition for Ivana and Tork, and insertion followed by elimination of new copies for Tekay. Whereas CRM and Tekay elements concentrate in centromeric and pericentromeric regions (see above), *Copia* elements Ivana and Tork have a more widespread chromosomal distribution (not shown) and our analysis shows that they are significantly enriched inside and near (5 kbp) genes in both species (Fig. 5b) and may therefore have impacted gene coding capacity and regulation, potentially triggering phenotypic consequences.

### LTR-retrotransposon TIPs impact on gene expression variability

**Fig. 6:**
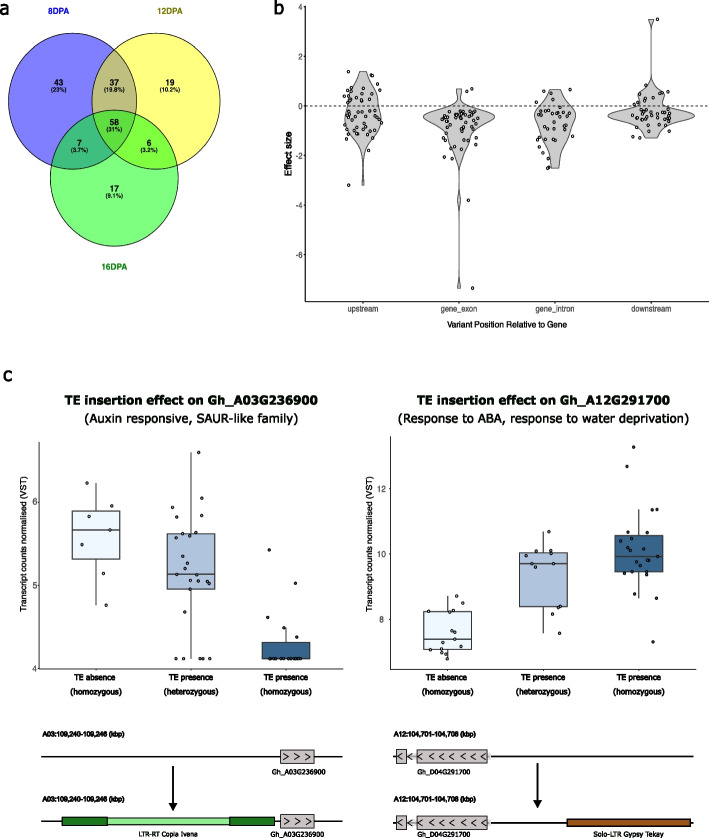
TIP-eQTLs found in a population of 50 G. hirsutum cultivars.** a** Venn diagram showing the number of TIP-eQTLs found in the three-fiber developmental timepoints analyzed (8, 12, and 16DPA). **b** Effect of the TE on gene transcription (beta values) divided by the TE position relative to the gene. **c** Examples of TIP-eQTLs: effect of each genotype on expression (VST) in each boxplot, accompanied by diagrams illustrating the gene and TE insertion relative positions

In order to assess the potential impact of TIPs close to genes on gene expression, we took advantage of a published fiber development expression dataset for *G. hirsutum* cultivated varieties [33]. We selected expression data from 50 varieties (Additional file 2: Table S5) for three timepoints of fiber development (8, 12, and 16 DPA, Days Post Anthesis) and performed TIP eQTL analyses. Among all TIPs characterized in *G. hirsutum*, 3854 were polymorphic (MAF > 5%) in the 50 selected varieties, and 1322 of these TIPs were located at less than 5 Kb of an annotated gene. We found a total of 187 TIP-eQTL significant associations across the three different fiber developmental stages (Additional file 2: Table S6). Out of these, 58 (31%) are present in all three stages, and 50 TIP-eQTLs (26%) are in two of the stages (Fig. 6a), which is to be expected as many genes are activated across more than one developmental stage [33]. Functional enrichment analysis of these genes shows significant enrichment for key functional categories such as “response to auxin” (GO:0009733) and “auxin-activated signaling pathway” (GO:0009734), as well as other functions linked to fatty acid metabolism and elongation (GO:0016620, GO:0009084). These functions have previously been associated with cell wall deposition and fiber elongation [34, 35]. As expected, the effect of the insertions on gene expression is, in most cases, negative (80%), in particular for insertions within coding regions (Fig. 6b). Nonetheless, insertions that result in gene activation also exist, in particular for insertions in the upstream and downstream proximal regions (Fig. 6b and c for particular examples). An analysis of the polymorphic insertions linked to changes in gene expression shows that, as expected, most of them belong to the Tork (60%) and Ivana (18%) lineages, although there are also some solo-LTR TIPs of *Gypsy* elements (11%). These results show that close insertions of LTR-retrotransposons can influence the expression of genes involved in fiber development, an agronomically important process, and suggest that TIPs close to genes could have an important impact on gene expression variability in this species.

### LTR-retrotransposon TIPs impact on cotton domestication and breeding

To address the possibility that genomic variability triggered by recent TIPs had an impact on cotton domestication and improvement, we analyzed their presence in wild and domesticated *G. hirsutum* and *G. barbadense* populations, including landraces and elite varieties. To this end, we mapped short-read resequencing data of 283 varieties of *G. hirsutum* and 223 varieties of *G. barbadense* to their respective pangenome graphs and genotyped the presence/absence of the 6841 *G. hirsutum* and 5749 *G. barbadense* TIPs across these populations. The varieties analyzed represent the wild-to-domesticated continuum of both species, including 151 and 51 elite varieties, 66 and 60 landraces, and 73 and 112 wild accessions of *G. hirsutum* and *G. barbadense,* respectively (Additional file 2: Tables S7a, S7b) [27].

**Fig. 7:**
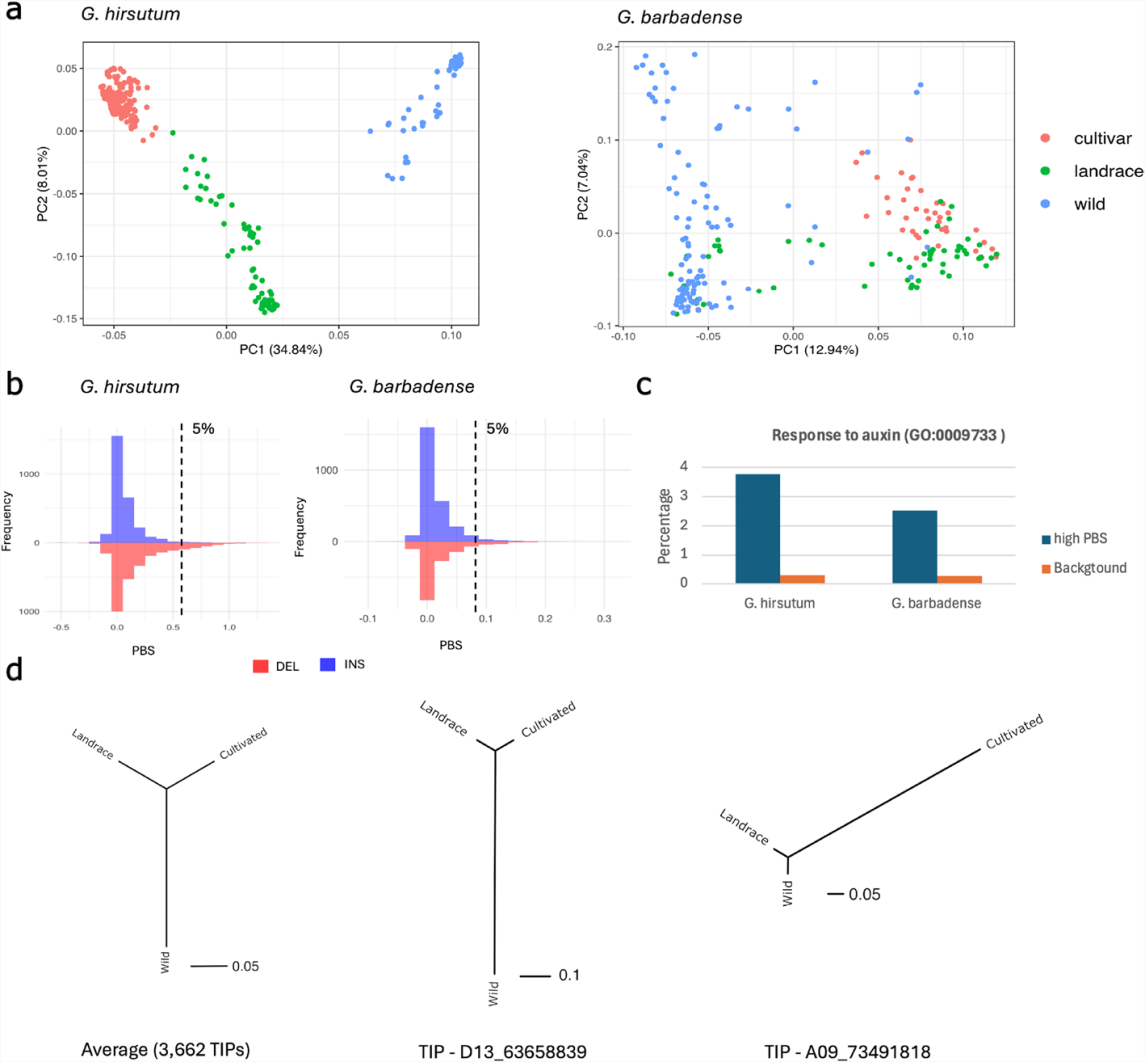
Changes in TIP frequencies accompanying *G. hirsutum* and *G. barbadense* domestication and breeding. **a** PCA based on TIPs in wild, landrace and domesticated varieties of *G. hirsutum* and *G. barbadense*. **b** Distribution of Population Branch Statistic values for TIPs (deletions and insertions with respect to the reference genome) for the comparison of cultivated against landrace and wild accessions in *G. hirsutum.* and *G. barbadense*. **c** Percentage of auxin-response GO term in the group of genes linked to a TIP with high PBS (5% top PBS) compared with the percentage of this GO term in the whole *G. hirsutum* proteome. **d** Fst-based tree representing the distance between the three populations using all shared TIPs (*n* = 5535), a deletion TIP 565 bp upstream of a SAUR-like gene (Gh_D13G264300, auxin-responsive gene) potentially related with domestication (D13:63,658,839) and a deletion TIP present in the intron of a SAUR-like gene (Gh_A09G167200, auxin-responsive gene) potentially related with cotton diversification

Principal Component Analysis (PCA) shows that TIPs provide enough signal to distinguish between groups of wild, landrace, and domesticated varieties and suggests that there has been extensive differential transposition activity and/or retention/elimination of LTR-retrotransposon copies and solo-LTRs between the three groups of accessions (Fig. 7a). This is particularly clear for *G. hirsutum*, in line with previous analyses based on SNPs, which showed that the cultivated *G. hirsutum* accessions clustered tightly due to their narrow genetic diversity [27]. This drastic reduction of diversity accompanying domestication was not seen in *G. barbadense*, probably because modern *G. barbadense* cultivars have a more complex and obscure origin that, in addition, involves many intentional introgressions [27].

An analysis of the TIP frequency in G*. hirsutum* and *G. barbadense* populations shows that there are few LTR-retrotransposon insertions or deletions present in only one or two groups (see Additional file 2: Table S8), whereas most TIPs (5860 in *G. hirsutum* and 4450 in *G. barbadense*) are present in all three groups (i.e., wild, landrace, and domesticated). Interestingly, however, the TIPs shared by the three groups are present in very different frequencies among accessions in those groups (Additional file 1: Figs. S6, S7). Although the presence of TIPs at different population frequencies may be due to founder effects or differences in demographic histories among these groups, these differences could also reflect a history of selection, positive or purifying, acting on polymorphic LTR-retrotransposons or on closely linked *loci*. To find signs indicative of positive selection of TIPs in cultivated varieties, we used the population branch statistics (PBS) method. This approach measures the level of genetic differentiation for a specific population along a phylogenetic branch, providing insights into population-specific evolutionary changes [36]. Figure 7b shows the PBS distribution of LTR-retrotransposon insertions and deletions relative to the reference genome in cultivated versus landrace and wild accessions and in wild versus cultivated and landrace accessions in *G. hirsutum.* We identified the TIPs with the highest PBS (above the 95th percentile in the comparisons cultivated against landraces and wild, and wild against cultivated and landraces). Eighty percent of the high-PBS TIPs of the wild versus cultivated and landraces (linked to domestication) and 63% of high-PBS TIPs in cultivated versus wild and landraces (linked to cotton breeding) are related to *Gypsy* LTR-retrotransposon (mainly Tekay) elements (Additional file 2: Table S9). Tekay elements are concentrated in the pericentromeric region of chromosomes, particularly in the D subgenomes, and our results show that they have contributed to the recent evolution of these regions, as discussed above. Many of the Tekay TIPs with high PBS are far from genes (Additional File 2: Table S9), have a distribution similar to the Tekay intact elements (Fig. 4 and Additional file 1: Fig. S5b), and are in regions characterized by a low density of coding genes. The PBS results presented here may suggest that Tekay insertions in these regions may have been directly or indirectly selected during domestication and improvement of both *G. hirsutum* and *G. barbadense*, although the confounding effects of population structure and demographic histories need to be considered, as already highlighted.

Although most high-PBS Tekay TIPs are far from genes, others are located close to genes (less than 2 Kb away, Additional file 2: Table S8 and Additional file 1: Fig. S4b) and may have impacted their coding capacity or regulation. This is also the case for high-PBS TIPs related to *Copia* LTR-retrotransposons, mainly the Ivana and Tork superfamilies, which are frequently found tightly associated with genes. The high PBS associated with these TIPs may suggest selection due to favorable or detrimental effects on the nearby genes. We analyzed the expression of genes tightly linked with high-PBS TIPs using previously published data for the *G. hirsutum* TM1 accession [37] and found that they show different patterns of expression throughout cotton development and under different stress conditions (Additional file 2: Table S8). An analysis of the functions of these genes shows a significant enrichment for “auxin response” genes (*p*-value = 0.00039, GO term GO:0009733) in *G. hirsutum*. We found 5 genes belonging to this functional category (four coding for SAUR-like auxin-responsive proteins and one for an Indoleacetic acid-induced protein 16) within the 95 percentile of highest PBS in *G. hirsutum,* indicative of high population frequency differentiation. This represents an 11-fold enrichment in comparison to the total annotated predicted genes (Fig. 7c). The PBS analysis of two of these TIPs is represented in Fig. 7d, as examples. For the first of these (D13_63658839), we observed a strong population frequency differentiation of the landraces and cultivated accessions with respect to the wild (potential relationship with domestication), whereas for the second (A09_73491818), we found a strong differentiation between the cultivated and the other two groups, suggesting that this TIP could have been targeted during crop improvement. A preliminary analysis of the TIPs with high PBS values in *G. barbadense* TIPs also showed an enrichment for the function “response to auxin” (*p* = 0.022) (Fig. 7d), suggesting that variants in auxin-related genes were selected in the domestication and breeding process of the two species of cotton, with the same caveat mentioned above, namely, that selection may mimic the effects of population structure and demography for any single gene. Whether this result reflects parallel selection or historical interspecific introgression is at present an open question.

## Discussion

Plant TEs, and in particular LTR-retrotransposons, have been traditionally assumed to evolve through bursts of amplification followed by periods of low activity and elimination [11]. Recent data, however, suggest that the diversity of LTR-retrotransposons that coexist within plant genomes often results from heterogeneous evolutionary dynamics [12]. For this study, we selected high-quality genome assemblies (at pseudo-chromosome level) of diploid and tetraploid species of cotton for an in-depth LTR-retrotransposon comparative analysis. Although we cannot completely rule out biases due to differences in assembly quality, the similar numbers of LTR-retrotransposons observed within species for each lineage support the robustness of these assemblies. The results presented here show that LTR-retrotransposon elements are in general more abundant and recent in the two tetraploid cotton species compared with models of their diploid progenitors, suggesting a general burst of amplification accompanying the polyploidization event, as it is often but not always the case in plants [9]. However, these results also show that different LTR-retrotransposon lineages experienced rather different evolutionary dynamics during cotton polyploidization, speciation, and subsequent crop improvement processes. As an example, whereas Ogre elements amplified in the tetraploids, they probably became silent before speciation, as they show little polymorphism between the two tetraploid species. Other LTR-retrotransposon lineages, such as Tork and Ivana, have probably retained activity for longer, since more than half of their insertions are polymorphic within the species. This is also the case for CRM and Tekay, which have recently been proposed to play important roles in the evolution of *Gossypium* centromeres [30, 31].

Our results show that CRM elements amplified after polyploidization. This CRM element number increase has been higher in the A than in the D subgenomes, probably because they are more efficiently removed from the latter subgenome, as evidenced by a higher number of solo-LTRs. As CRMs concentrate in cotton centromeric regions, this leads to a differential increase of CRMs in the centromeric regions of the A subgenomes in both tetraploid species relative to the model diploid progenitor genome (represented by modern *G. herbaceum*). Interestingly, the number of CRM solo-LTRs in the D subgenomes is higher in *G. barbadense* than in *G. hirsutum*, congruent with the lower number of intact CRMs in this species, suggesting that the rate of centromeric CRM elimination in the D subgenomes is higher in this species. In contrast to CRM LTR-retrotransposons, Tekay elements have greatly increased in the D subgenomes while their number has remained constant in the A subgenomes. These results are in line with recent evidence regarding the role of these two lineages in centromere evolution in cotton after polyploidization, with the proportion of CRM versus Tekay elements being higher in the centromeric regions of the A subgenomes, and lower in those of the D subgenomes [30]. Our results add to these earlier observations in suggesting that this shift may reflect differences in elimination of the insertions between the two subgenomes rather than differential insertion. Moreover, the analysis of CRM and Tekay intact and potential solo-LTR distributions along chromosomes suggests that, although these elements may be centrophilic [38] and target the centromere for integration, their elimination via solo-LTR formation from chromosome arms reinforces their concentration in centromeric and pericentromeric regions. The observed pattern is likely also influenced by the well-known suppression of homologous recombination in centromeric regions, which will also limit the formation of solo-LTRs via unequal recombination. Taken together, our results are compatible with the important role of CRM and Tekay LTR-retrotransposons in the expansion and reorganization of cotton centromeres after polyploidization that has been recently suggested [30, 31] and point to the role of their selective elimination from chromosomal regions and subgenomes in specifying their dynamics and their genomic distribution.

Interestingly, our results suggest greater transposable element turnover after polyploidization in cotton than previously reported. Some of our observations are congruent with previous studies, specifically the aforementioned change in relative centromere composition, as well as our observation of post-polyploidization expansions of some lineages of LTR-retrotransposons, particularly in the D subgenome [30, 39]. Conversely, our results suggest far greater transposable element activity after polyploidization than earlier reports [39], which used phylogenetic analysis of PCR-amplified reverse transcriptase domains to find little evidence of impressive proliferation associated with polyploidy. Here we find elements that exhibit rapid bursts associated with polyploidization (e.g., Ogre), becoming silenced sometime either immediately after polyploidization or prior to the divergence of *G. hirsutum* and *G. barbadense*, as well as elements that show evidence of ongoing activity in one or both of the species evaluated here. The abundance of TIPs, particularly solo-LTR TIPs, suggests that transposable element proliferation after polyploidization has been common and differential among species and subgenomes for some element families, and thus, there has been historical and ongoing transposition in polyploid cotton.

With respect to continuing LTR-retrotransposon activity within species, our results show that CRM and especially Tekay intact LTR-retrotransposon and solo-LTR insertions are highly polymorphic within the two polyploid species, and are present at different population frequencies in wild, landrace, and cultivar groups of accessions. Interestingly, many of the Tekay TIPs are among those with the highest Population Branch Statistic (PBS) values, which may indicate potential selection during domestication and breeding, although selection is difficult to demonstrate convincingly using exclusively this type of analysis, as spurious associations may also result from population structure and bottlenecks. The intriguing possibility of selection potentially operating on pericentromeric Tekay TIPs raises the possibility of their role in generating phenotypic variation visible to selection. Centromeres are fast-evolving structures [32], and it has previously been shown that centromere variants have been selected during the domestication of crops, such as maize and wheat [40, 41]. Recently, repositioning of a centromere has also been documented for the domesticated *G. hirsutum* accession ZM113 [42], providing evidence for the potential impacts of domestication on centromere evolution in cotton. The putative selection on centromeric variants suggested here could also reflect selection on linked genes residing in this low recombining region, although, as noted above, associations indicative of selection may also result from population structure and bottlenecks accompanying crop improvement. Disentangling the relative impacts of the foregoing forces will require further analyses.

In addition to being important players in centromere evolution [32], LTR-retrotransposons have also played a major role as drivers of genetic diversity in plant gene coding capacity and regulation [2, 43]. Our data show that, in addition to *Gypsy* CRM and Tekay, two *Copia* LTR-retrotransposon lineages (Tork and Ivana) have amplified and remained active in the two polyploid species of cotton since their divergence from a common ancestor. Notably, we identified a large number of recent retrotranspositions, as evidenced by their identical LTRs, that are significantly enriched within exons and in the proximal regions upstream and downstream of genes. A TIP-eQTL analysis on 50 cultivated varieties of *G. hirsutum* shows that TIPs within or close to genes can impact the expression of fiber-development genes and points to a relevant impact of TIPs located close to genes on gene expression in cotton.

For many of the TIPs characterized here, frequencies in the wild, landrace, and cultivar groups vary widely, exhibiting high PBS values and suggesting the possibility that selection during cotton domestication and crop improvement may have shaped their distribution. Gene expression data reveals a wide range of developmental and stress-related expression patterns. Considering the many different traits targeted by domestication and crop improvement, including plant architecture, fruiting habit, flower and seed development, and fiber length and quality [44], additional work is needed to determine whether the genomic changes introduced by these genic and gene-adjacent LTR-retrotransposon insertions have contributed to potentially selectable trait variation. It is interesting to note that both *G. hirsutum* and *G. barbadense* exhibited an overrepresentation of auxin-responsive genes, which include SAUR-like genes, among those impacted by LTR-retrotransposon insertions (Additional file 1: Fig. S8). Auxins are well-established as key controllers of plant development and stress responses [45], which also play a role in the regulation of cotton fiber development [46, 47] and broader processes such as plant growth [48, 49]. Moreover, SAUR genes, which are key players in plant adaptation and growth [50], have been shown to have been targets of domestication and breeding of crops such as rice, citrus, and *Brassica oleracea* [51–53], and a subset of the auxin-responsive SAUR-like gene family has been observed to be involved in cotton fiber development [54]. The potential independent selection of transposon insertion polymorphisms closely linked to different auxin-related genes in both *G. hirsutum* and *G. barbadense* highlights the importance of this hormone signaling in cotton domestication and breeding and suggests convergent selection in both species (notwithstanding the possibility of an interspecific introgressive origin).

## Conclusions

This study highlights the importance of considering lineage-level TE classifications when studying LTR-retrotransposon dynamics, as well as examining not only retrotransposition but also recombination and the formation of solo-LTRs as the joint proximal determinants of TE presence in genomes. Our study shows that LTR-retrotransposon dynamics are highly differential among LTR-retrotransposon families, and that only some were activated after polyploidization and disparately between the two cotton species and the two subgenomes in each. CRM and Tekay elements appear to have played key roles in centromere reorganization, and Tork and Ivana are suggested to actively generate variability within and close to genes. These few recently active lineages have generated LTR-retrotransposon polymorphisms that may have been selected during cotton domestication and crop improvement, have modified chromosome heterochromatic regions, and have impacted genes expressed in different tissues and environmental situations, including genes related to auxin signaling, which are key players in plant and fiber development regulation, in both *G. hirsutum* and *G. barbadense*.

## Methods

All scripts and pipelines used for this study are available within the following directory: https://github.com/Lcamdom/panTEvo [55].

### Annotation of intact LTR-retrotransposons and estimation of insertion times

All genomes selected and included in this study (Additional file 2: Table S1) have been assembled into pseudo-chromosomes, and only these sequences were used in our analysis. These were annotated for intact LTR-retrotransposon elements using EDTA-raw scripts on the LTR-mode using default parameters [56]. The intact elements found were further classified using TEsorter on default parameters and with the rexdb-plant database [57]. The insertion times were estimated by EDTA (which uses LTR-retriever for this); they are calculated by multiplying the divergence between the two LTR sequences by the neutral mutation rate of rice [57].

The phylogenetic analysis of RT sequences was performed by extracting the RT domains of CRM and Tekay elements from the TEsorter output, filtering it by 90% coverage and clustering it using cd-hit [58] to eliminate redundancy. Sequences present in the genome assemblies of *G. hirsutum*, *G. raimondii*, and *G. herbaceum*, showing 80% sequence identity over 90% query coverage with these RT sequences, were identified by Blast [59]. Resulting hits were processed using bedtools [60], and RT protein domains were annotated using TEsorter again. The obtained protein sequences were aligned using mafft (–auto), trimmed using trimal default parameters [61], and Maximum Likelihood phylogenetic trees were generated using FastTreeMP (-lg -fastest -gamma) [62]. The tree visualization and representation were performed using iTOL [63].

Density of intact Tekay and CRM elements was represented along the genome of *G. herbaceum*, *G. raimondii*, *G. hirsutum* (TM1), and *G. barbadense* (Pima90) using the R package KaryoplotR [64].

### Annotation of solo-LTRs

All intact elements within the same species and lineage were clustered using CD-hit [58] for 80% similarity and 80% coverage. The representatives for each cluster were used to build species-specific, home-made in silico libraries of intact LTR-retrotransposon elements. For each element in these libraries, the left LTR was extracted using the structural annotation generated by LTR_Retriever [65], and species-specific home-made libraries of LTRs and LTR-retrotransposon internal (INT) regions were generated. Solo-LTRs were then identified in each genome using these libraries. We did this by looking for sequences showing 80% sequence identity over 80% of the query length using Blast [59] with the LTR library as query. We then characterized the solo-LTRs by filtering out any LTR hit with any LTR-retrotransposon internal (INT) sequence within 1 kb either upstream of the start or downstream of the end.

Density of solo-LTRs from Tekay and CRM was represented along the genomes of *G. herbaceum*, *G. raimondii*, *G. hirsutum* (TM1), and *G. barbadense* (Pima90) using the R package KaryoploteR [64].

### Pangenome construction and TIP characterization

Minimap2 [66] was used to map every *G. hirsutum* and *G. barbadense* genome to the references (TM1 and 3–79, respectively, all assemblies used in Stable 1), using default parameters and low divergence setting (asm5). Structural variants (SVs) were called using svim-asm [67] with a minimum variant size of 100 bp, and the vcfs were merged using bcftools merge [68] and collapsed with truvari [69]. The pangenome graphs were generated using the vg toolkit [70].

TIPs were characterized using blast [59] to find homology between the pangenome SVs and the consensus elements from intact LTR-retrotransposon and solo-LTR libraries. Resulting hits were excluded when they represented less than 80% of the length of the TE consensus, had lower than 80% homology with the reference TE, and covered less than 80% of the SV. We identified potential TSDs associated with the TIPs by extracting 6-mers before and after the start and end coordinates of each Structural Variant, and searching for identical 5-mers between the 6-mers upstream of the SV and the last 6-mers of the SV and between the 6-mers downstream of the SV and the first 6-mers of the SV, allowing one mismatch between them.

### Pangenome genotyping and population frequency analysis

Re-sequencing data from 295 and 222 accessions across the wild-to-domesticated continuum (Additional file 2: Tables S6a and S6b) were mapped to the *G. hirsutum* and *G. barbadense* pangenomes, respectively. For each dataset, a subsample of 78 M reads was obtained (10 ×) for genotyping. Mapping of reads to the pangenome graph and structural variant calling were accomplished using the vg toolkit [70].

The TIP population frequencies were calculated using R. TIPs present in the three population groups were combined in a single matrix to obtain PBS values. TIPs with MAF > 0.01 and call rate > 95% were used to calculate Fst values using SNPready [71]. PBS values were calculated following the formulas described in [36]. GO enrichment of genes with proximal TIPs with high PBS was performed with GOATOOLS [72].

### Expression analysis of genes near high PBS TIPs

Available *G. hirsutum* RNA-seq raw reads [31] and assembled reference transcriptome [73] were processed to estimate transcript abundance using Trinity Transcript Quantification scripts [74] and Salmon [75] was used as the abundance estimation method. The raw count matrices were processed into Rlog values using DESeq2 [76]. Heatmaps are normalized per row (scale = rows), and were generated using the Pheatmap function in R.

### TIP-eQTL analysis

Re-sequencing datasets and RNA-reads (from 8, 12, and 16 DPA) from a population of 50 *G. hirsutum* individuals (Additional file 2: Table S5) [33] were used to perform TIP-eQTL analysis. The WGS datasets were used to genotype the TIP presence/absence along the genome in this population (see above pangenome genotyping section). We filtered the genotype matrix for TIPs with a Minimum Allele Frequency (MAF) of 5%. The RNA-seq data was used to estimate transcript abundance using Trinity and Salmon (see above expression analysis section). We used the count matrices to VST (Variant Stabilising Transformation) matrices for expression data using DeSeq2 [66]. We used Matrix eQTL [77] to find cis-TIPeQTLs with a maximum gene-TIP distance of 5 kbp.

## Supplementary Information

Additional file 1: Figure S1. N of copies and ages of intact elements and copies of solo-LTRs per lineage, genome and subgenome. Figure S2. Phylogenetic trees of LTR-RT sequences from Tekay and CRM elements in diploid and tetraploid cotton. Figure S3. Chromosomal distribution of CRM and Tekay elements in G. hirsutum and G. barbadense. Figure S4. Chromosomal distribution of CRM and Tekay elements in the parental diploids and G. hirsutum. Figure S5. SV and TIP distribution along the G. hirsutum genome. Figure S6. TIP population frequencies in G. hirsutum (a). Figure S7. TIP population frequencies in G. barbadense (b). Figure S8. Expression heatmaps of genes near (2kbp) high-PBS TIPs in G. hirsutum.

Additional file 2: Table S1. Genome assembly information table. Table S2. N of intact elements found per lineage, genome and subgenome. Table S3. N of TIPs classified by type and lineage in the G. hirsutum pangenome. Table S4. N of TIPs classified by type and lineage in the G. barbadense pangenome. Table S5. WGS and RNA-seq datasets used for eQTL analysis. Table S6. TIP-eQTL analysis results summary. Table S7. Dataset codes and accession names for G. hirsutum (a) and G. barbadense (b) pangenome genotyping. Table S8. TIPs shared across population groups in G. hirsutum and G. barbadense. Table S9. Summary of TIPs with high PBS in the G. hirsutum (a) and G. barbadense (b) pangenomes.

## Data Availability

The source code supporting this study is available in the GitHub repository https://github.com/Lcamdom/panTEvo.
