# Supplementary material for: Differential LTR-retrotransposon dynamics across polyploidization, speciation, domestication, and improvement of cotton (Gossypium)
Source: Genome Biol. 2025 Oct 27;26:369. doi: 10.1186/s13059-025-03837-7 (PMC12557855; doi:10.1186/s13059-025-03837-7)
Supplement: Supplementary file 1 — Additional file 1: Figure S1. N of copies and ages of intact elements and copies of solo-LTRs per lineage, genome and subgenome. Figure S2. Phylogenetic trees of LTR-RT sequences from Tekay and CRM elements in diploid and tetraploid cotton. Figure S3. Chromosomal distribution of CRM and Tekay elements in G. hirsutum and G. barbadense. Figure S4. Chromosomal distribution of CRM and Tekay elements in the parental diploids and G. hirsutum. Figure S5. SV and TIP distribution along the G. hirsutum genome. Figure S6. TIP population frequencies in G. hirsutum (a). Figure S7. TIP population frequencies in G. barbadense (b). Figure S8. Expression heatmaps of genes near (2kbp) high-PBS TIPs in G. hirsutum. [file 13059_2025_3837_MOESM1_ESM.pdf]

Figure S1. Intact LTR-RT and solo LTR content in diploid and tetraploid cotton by lineage and subgenome. Number of intact LTR-RT per megabase pairs per subgenome in each of the cotton species and varieties analyzed (top panels), violin plots representing the insertion times these intact LTR-RTs per subgenome in each of the cotton species and varieties analyzed (middle panels), and number of solo LTRs per megabase pairs per subgenome in each of the cotton species and varieties analyzed (bottom panels).

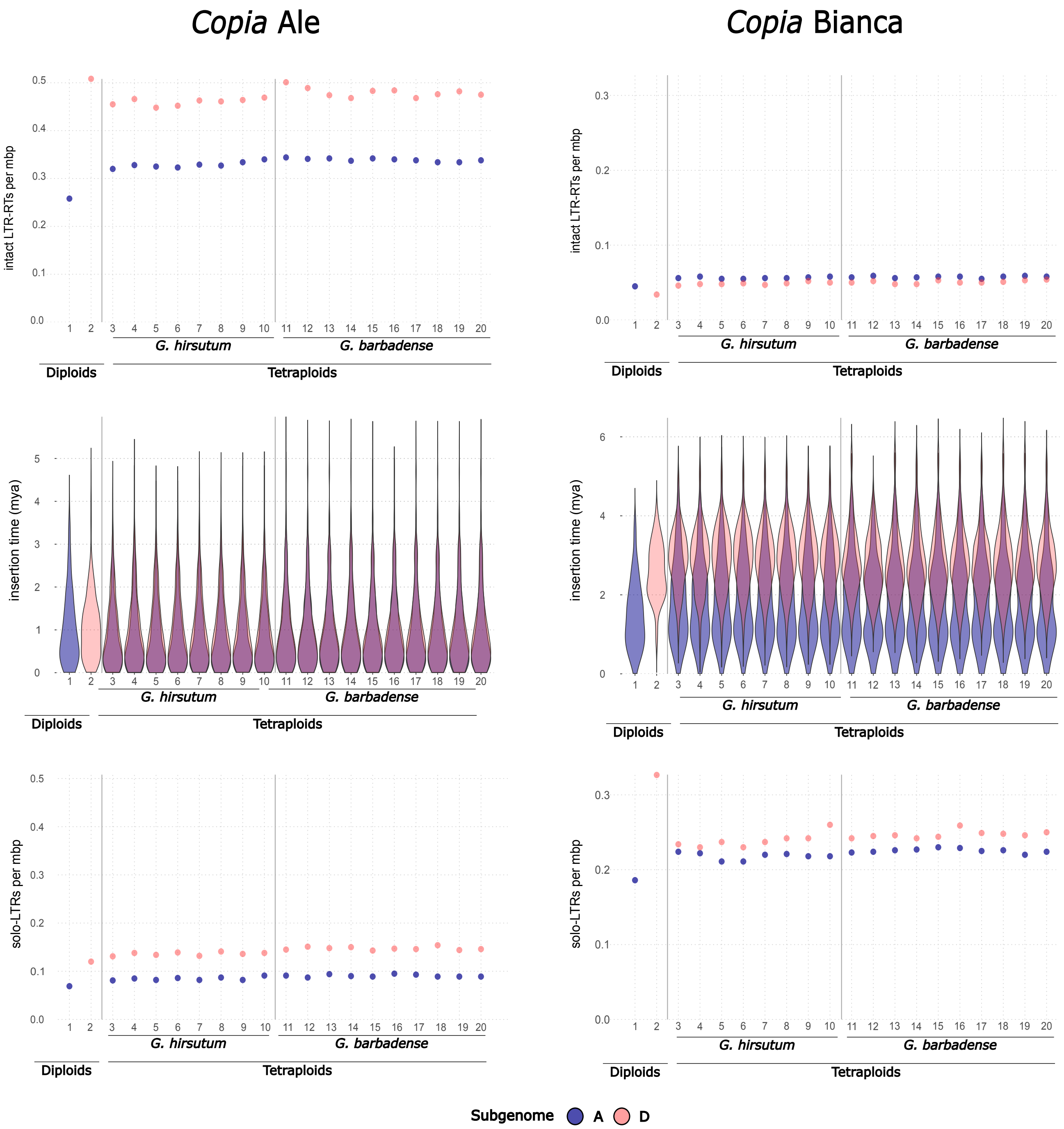

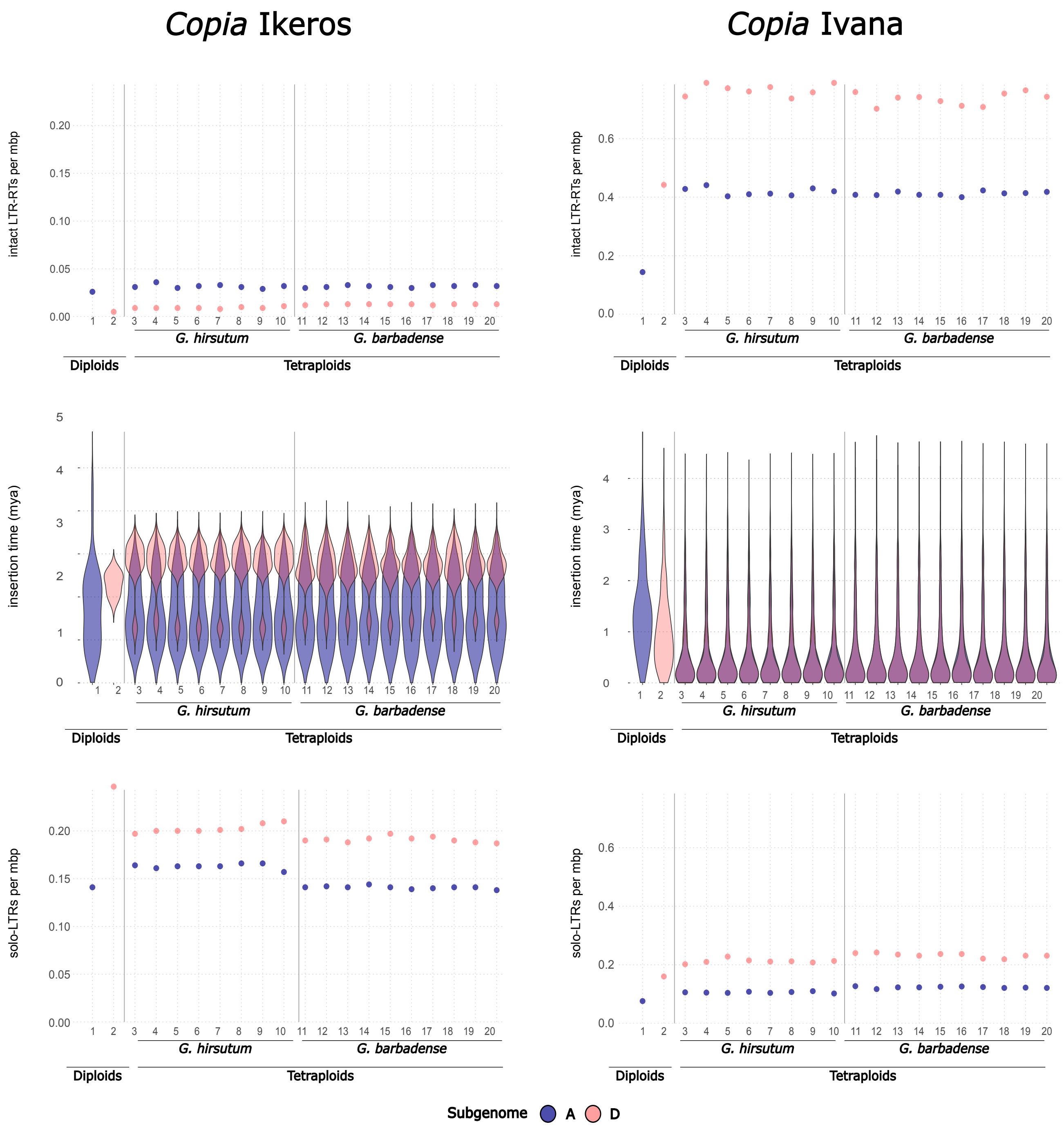
Copia Ikeros
Copia Ivana
intact LTR-RTs per mbp
insertion time (mya)
solo-LTRs per mbp
G. hirsutum
G. barbadense
Diploids
Tetraploids
Subgenome
A
D

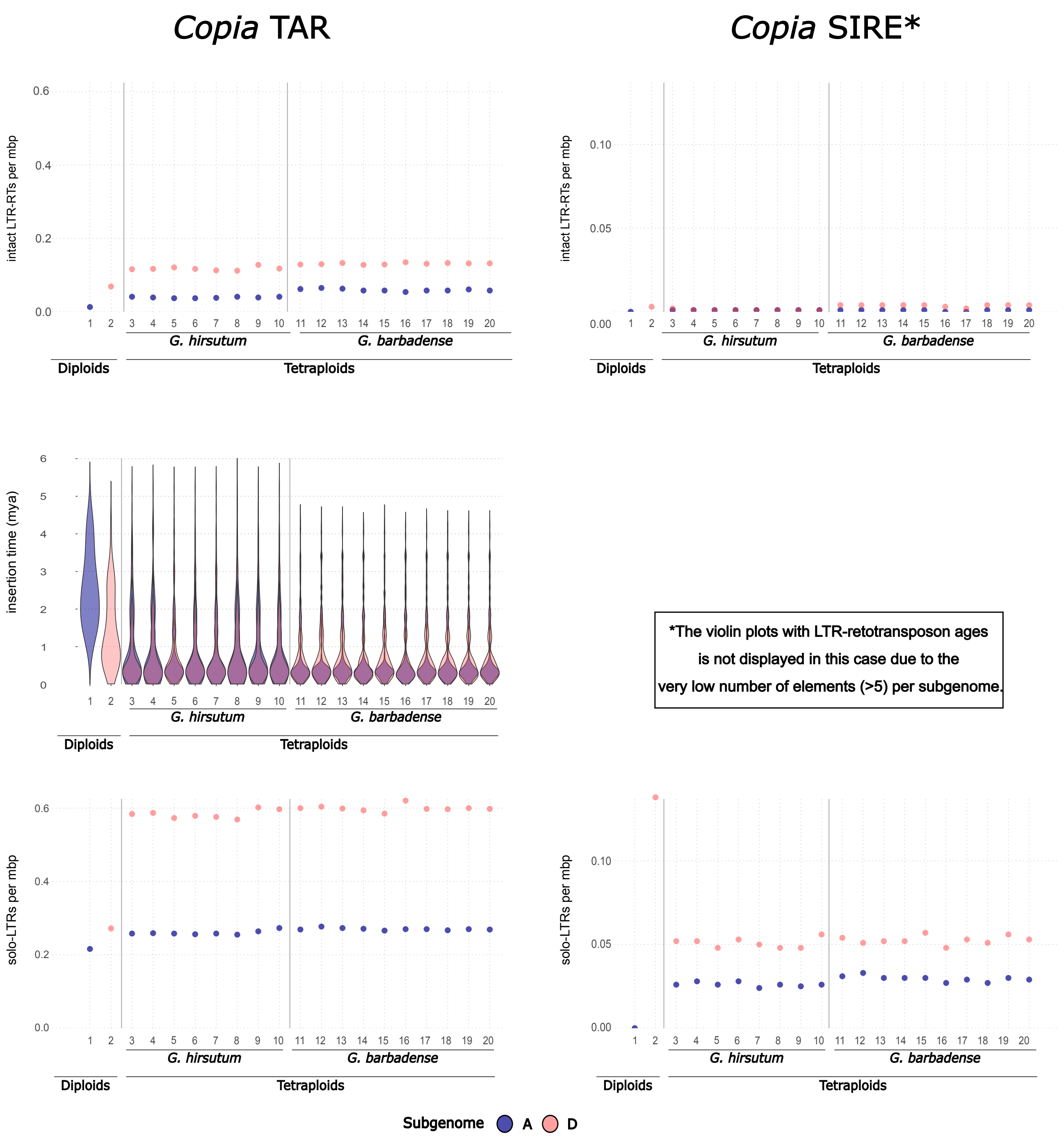

Copia TAR
Copia SIRE*
intact LTR-RTs per mbp
insertion time (mya)
solo-LTRs per mbp
1 2 3 4 5 6 7 8 9 10 11 12 13 14 15 16 17 18 19 20
G. hirsutum
G. barbadense
Diploids
Tetraploids
*The violin plots with LTR-retotransposon ages
is not displayed in this case due to the
very low number of elements (>5) per subgenome.
Subgenome A D

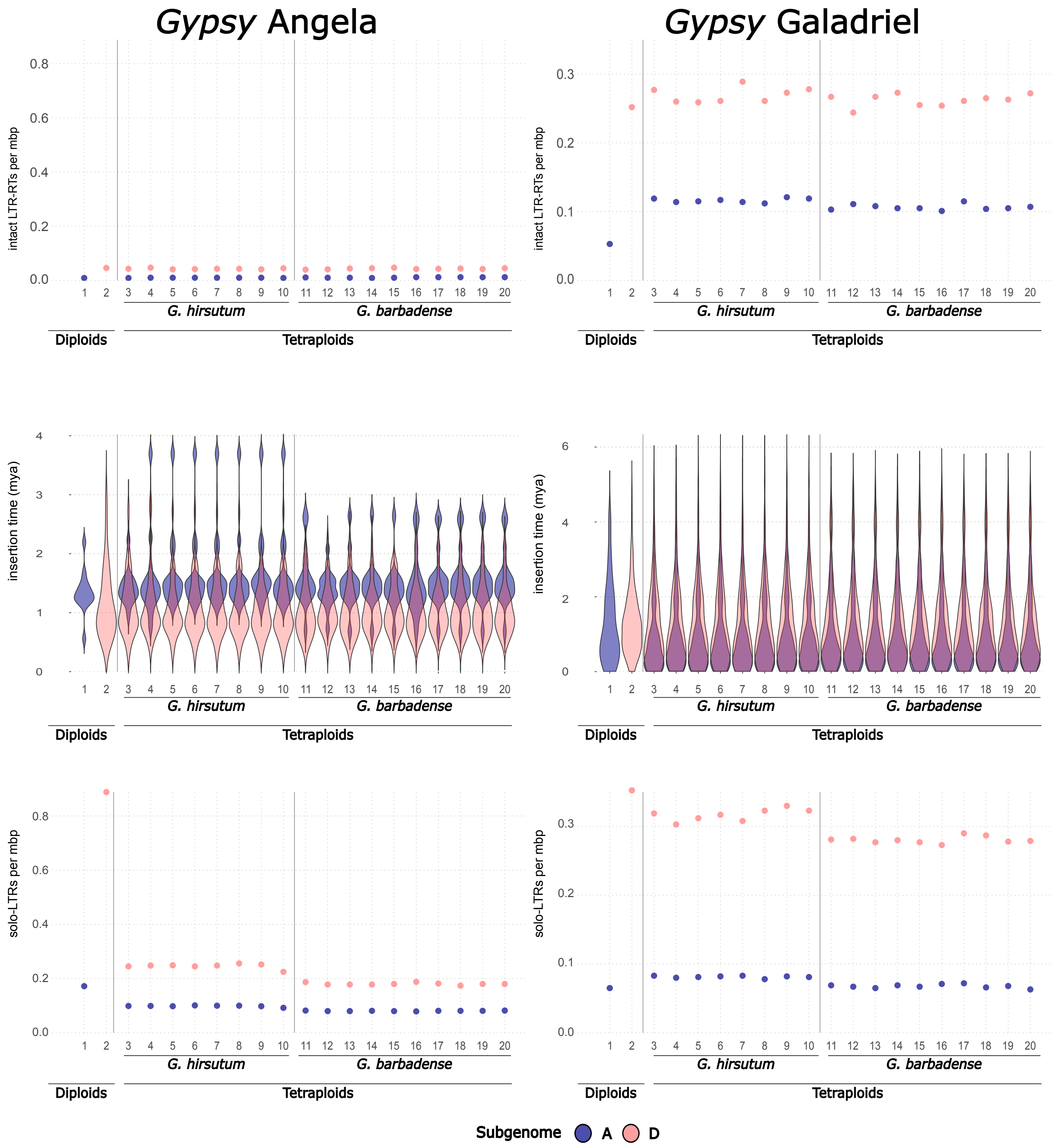
Gypsy Angela
Gypsy Galadriel
intact LTR-RTs per mbp
insertion time (mya)
solo-LTRs per mbp
1 2 3 4 5 6 7 8 9 10 11 12 13 14 15 16 17 18 19 20
G. hirsutum
G. barbadense
Diploids
Tetraploids
Subgenome
A
D

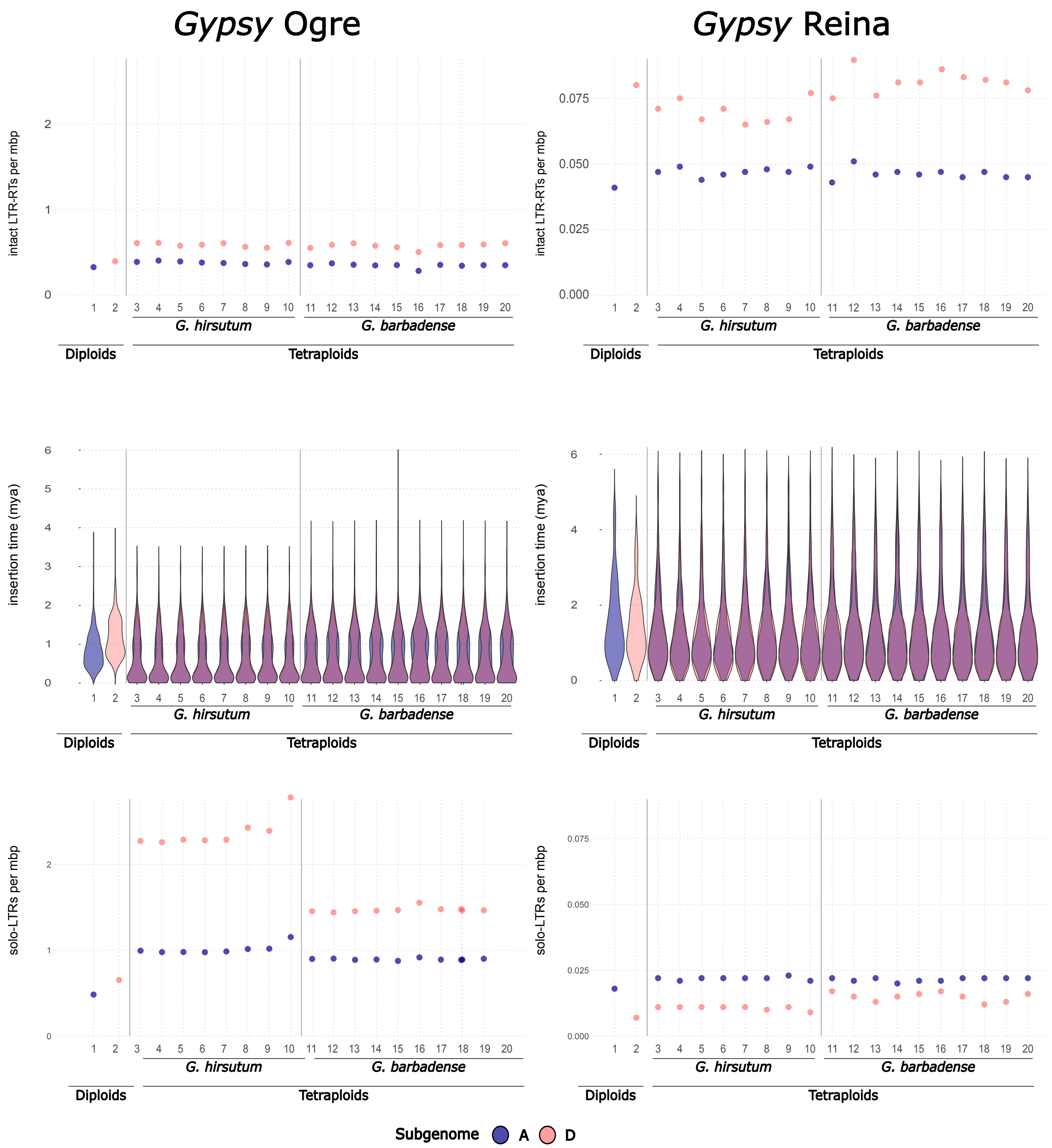
Gypsy Ogre
Gypsy Reina
intact LTR-RTs per mbp
insertion time (mya)
solo-LTRs per mbp
G. hirsutum
G. barbadense
Diploids
Tetraploids
Subgenome
A
D

Figure S2.Phylogenetic relationships between RT sequences from Tekay and CRM elements in diploid and tetraploid cotton. a) Maximum likelihood tree of 3,509 RT secuences from CRM elements of *G. herbaceum* (orange), *G. raimondii* (yellow) and *G. hirsutum* (green). b) Maximum likelihood tree of 64,659 RT secuences from Tekay elements of G. herbaceum (orange), G. raimondii (yellow) and G. hirsutum (green)

a

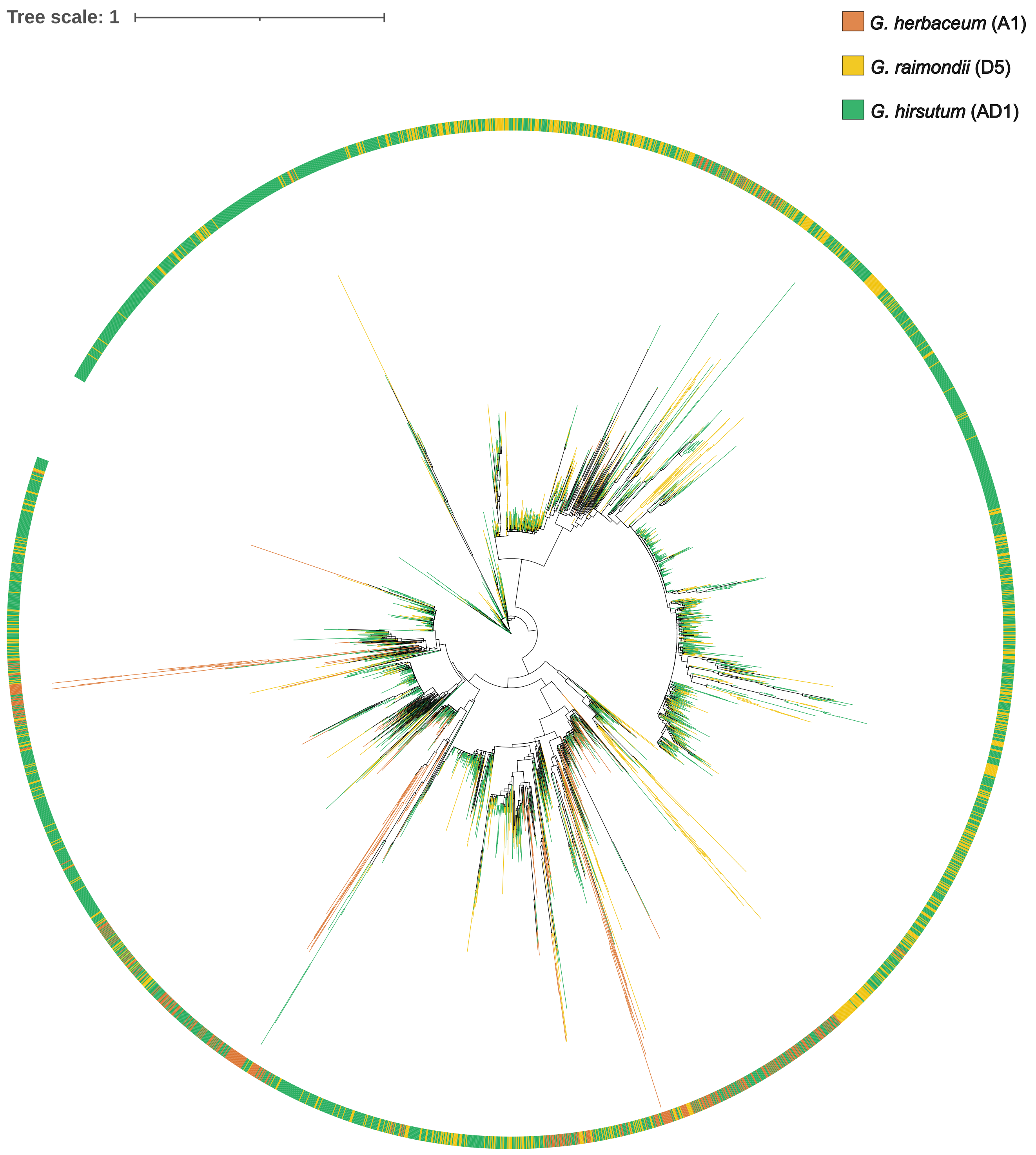

b

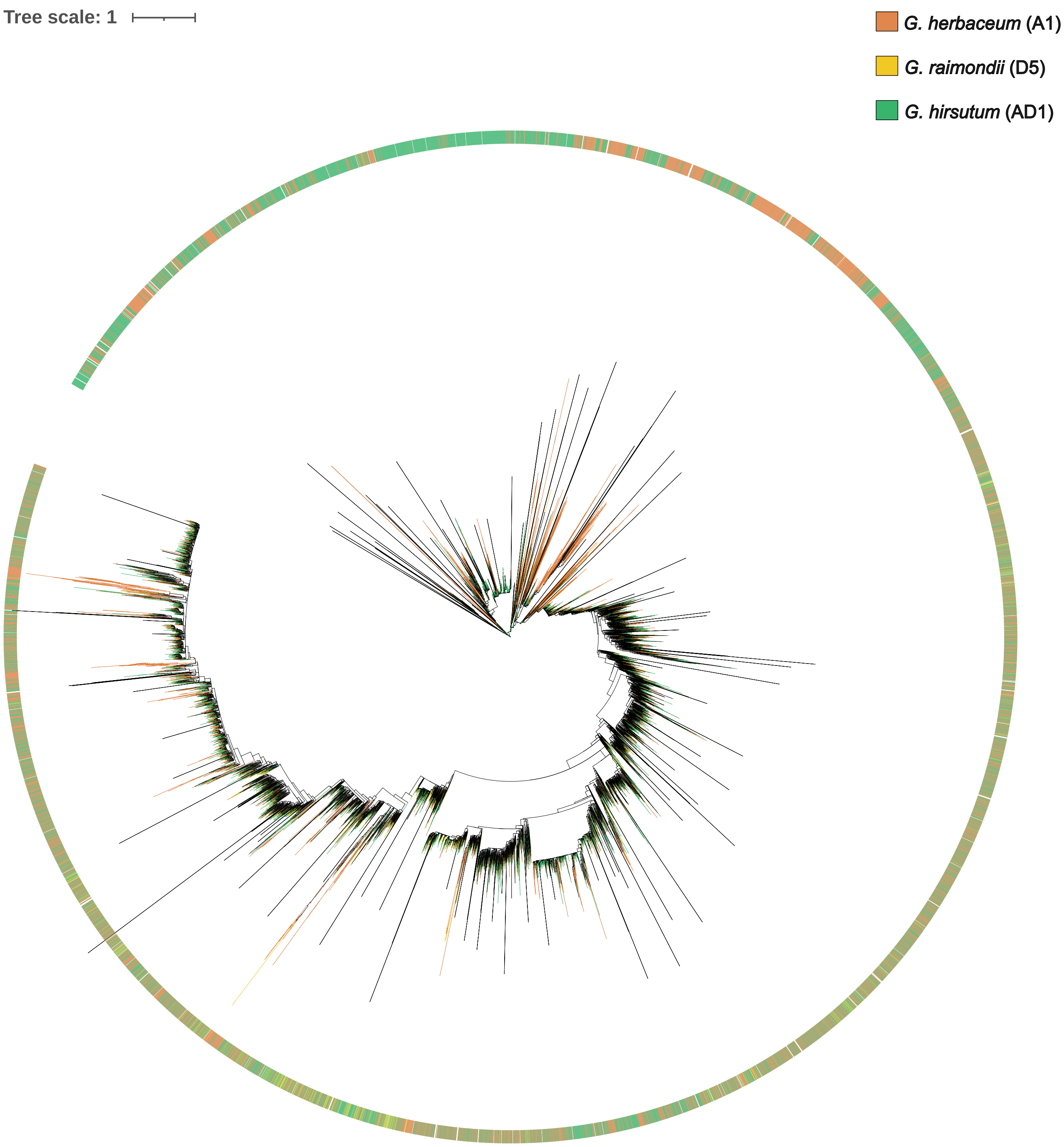

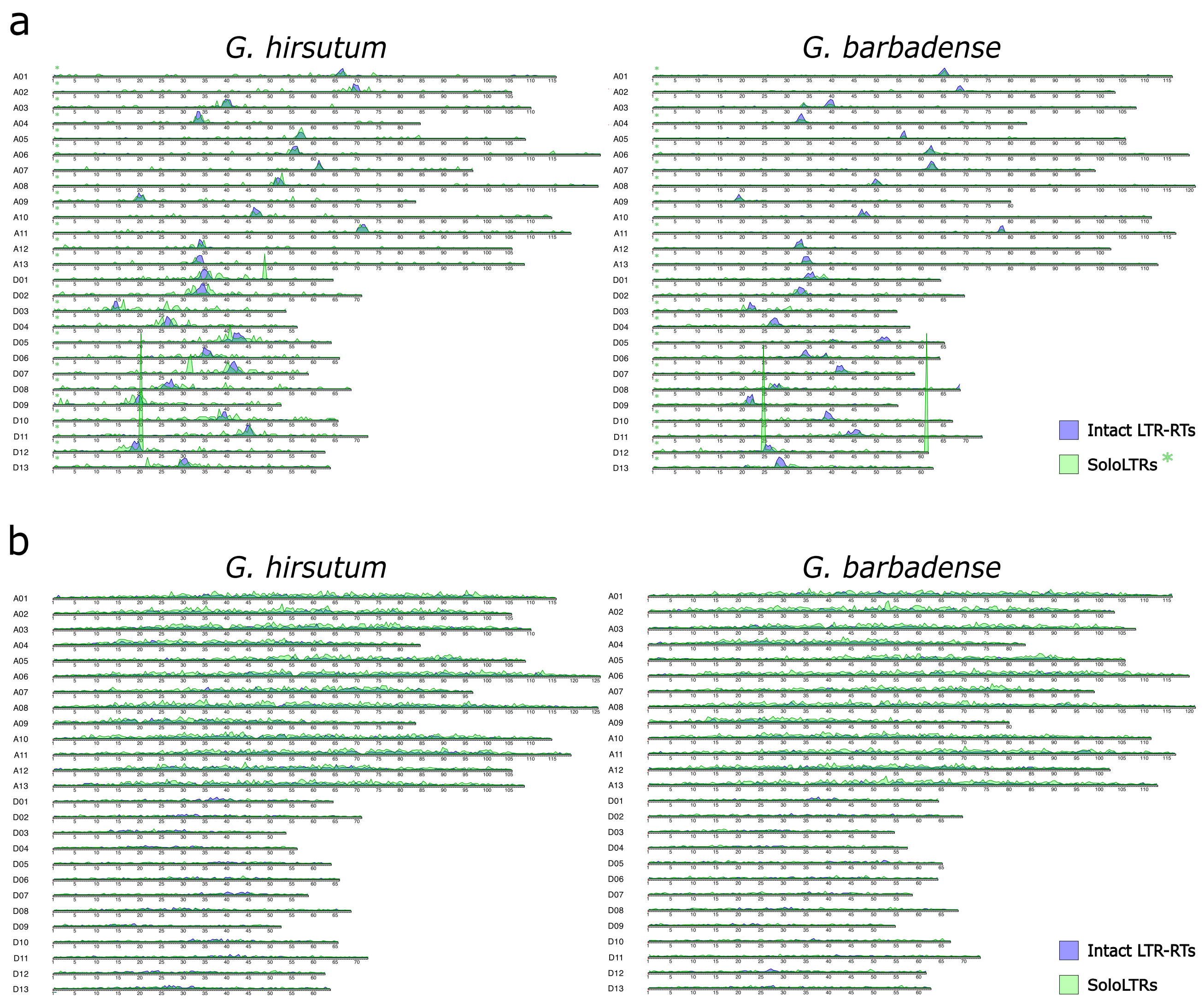

Figure S3. Chromosomal distribution of CRM and Tekay elements along the tetraploids *G. hirsutum* and *G. barbadense*. a) Density plots of CRM intact LTR-RTs and solo-LTRs along all chromosomes of *G. hirsutum* and *G. barbadense*. A green asterisk indicates where the density of solo-LTRs represented has been modified to make their distribution visible along chromosomes (scale of solo-LTRs is 10x the scale of intacts). b) Density plots of Tekay intact LTR-RTs and solo-LTRs along all chromosomes of *G. hirsutum* and G. *barbadense*.

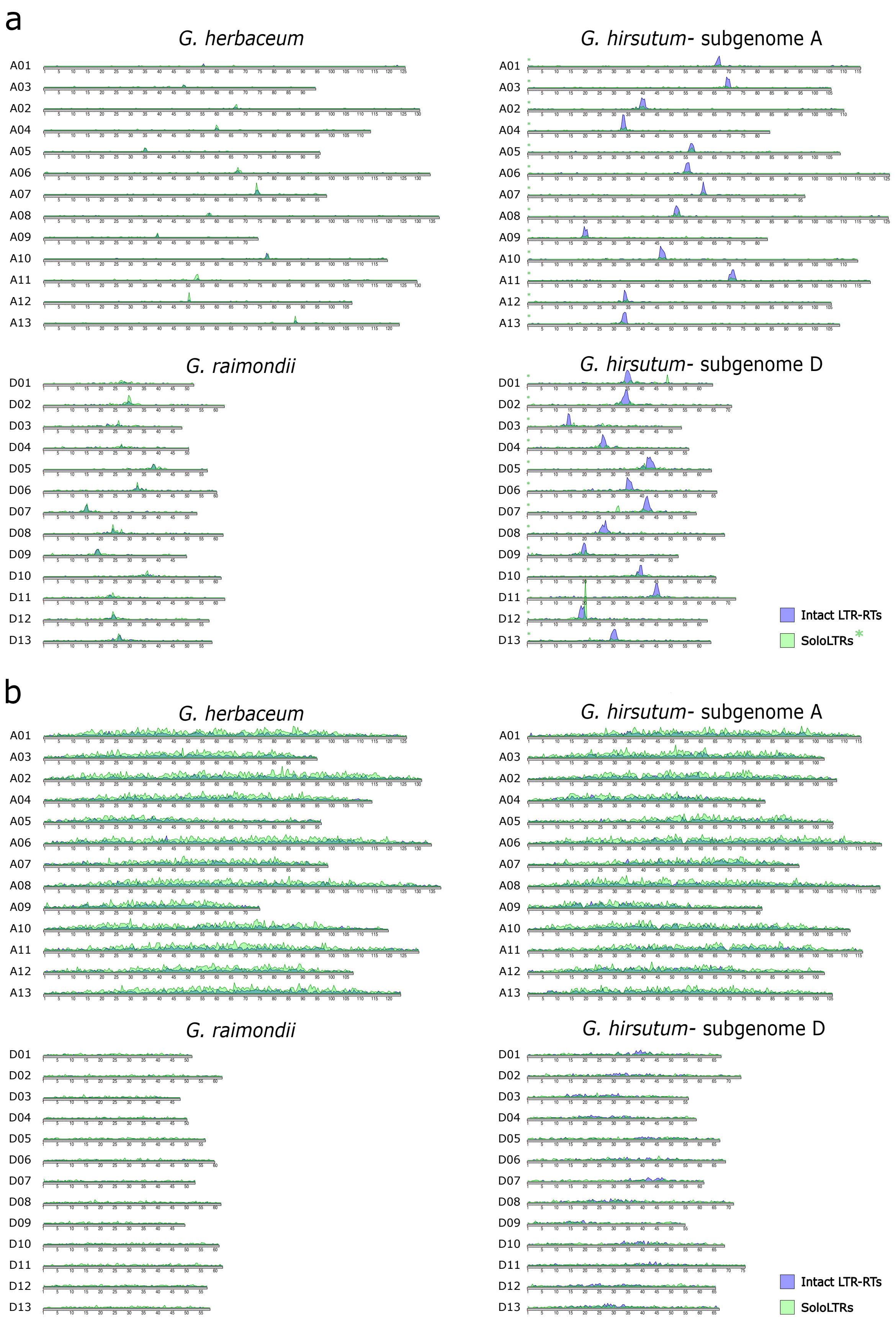

Figure S4. Chromosomal distribution of CRM and Tekay elements along the parental diploids *G. herbaceum* and *G. raimondii*, and the tetraploid *G. hirsutum*. a) Density plots of CRM intact LTR-RTs and solo-LTRs along all chromosomes of *G. herbaceum*, *G. raimondii* and *G. hirsutum*. A green asterisk indicates where the density of solo-LTRs represented has been modified to make their distribution visible along chromosomes (scale of solo-LTRs is 2.5x the scale of intacts). b) Density plots of Tekay intact LTR-RTs and solo-LTRs along all chromosomes of *G. herbaceum*, *G. raimondii* and *G. hirsutum*.

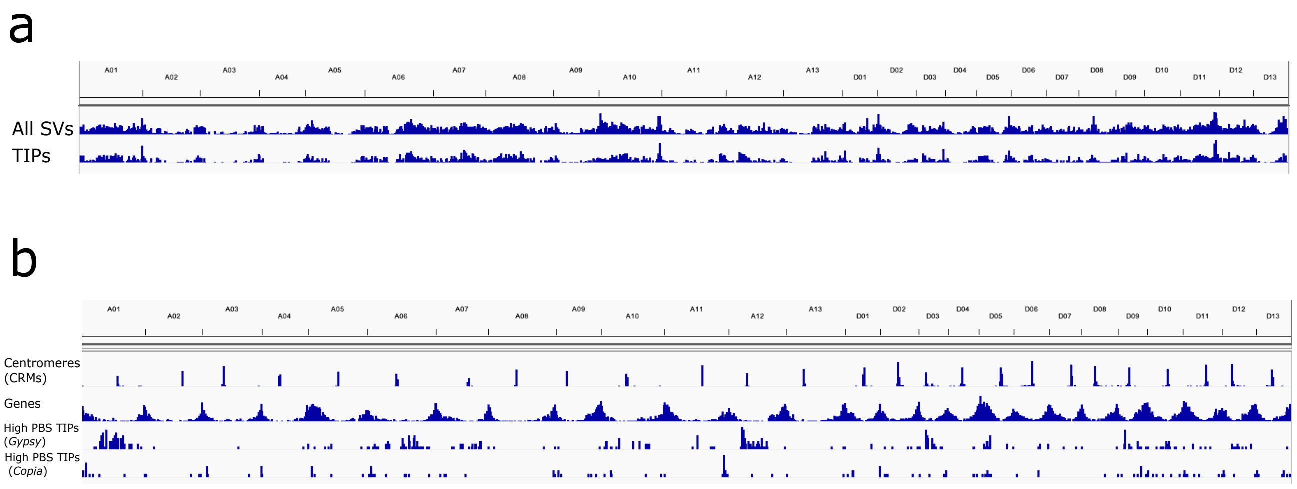

Figure S5. Distribution of SVs and TIPs along the *G. hirsutum* chromosomes. a) Distribution of SVs (top) and all TIPs (bottom) along the *G. hirsutum* chromosomes. b) Genome distribution of high PBS TIPs (split into *Gypsy* and *Copia* LTR superfamilies) along the *G. hirsutum* chromosomes (two bottom tracks). Centromere (intact CRM elements) and gene distribution in the two top tracks for reference.

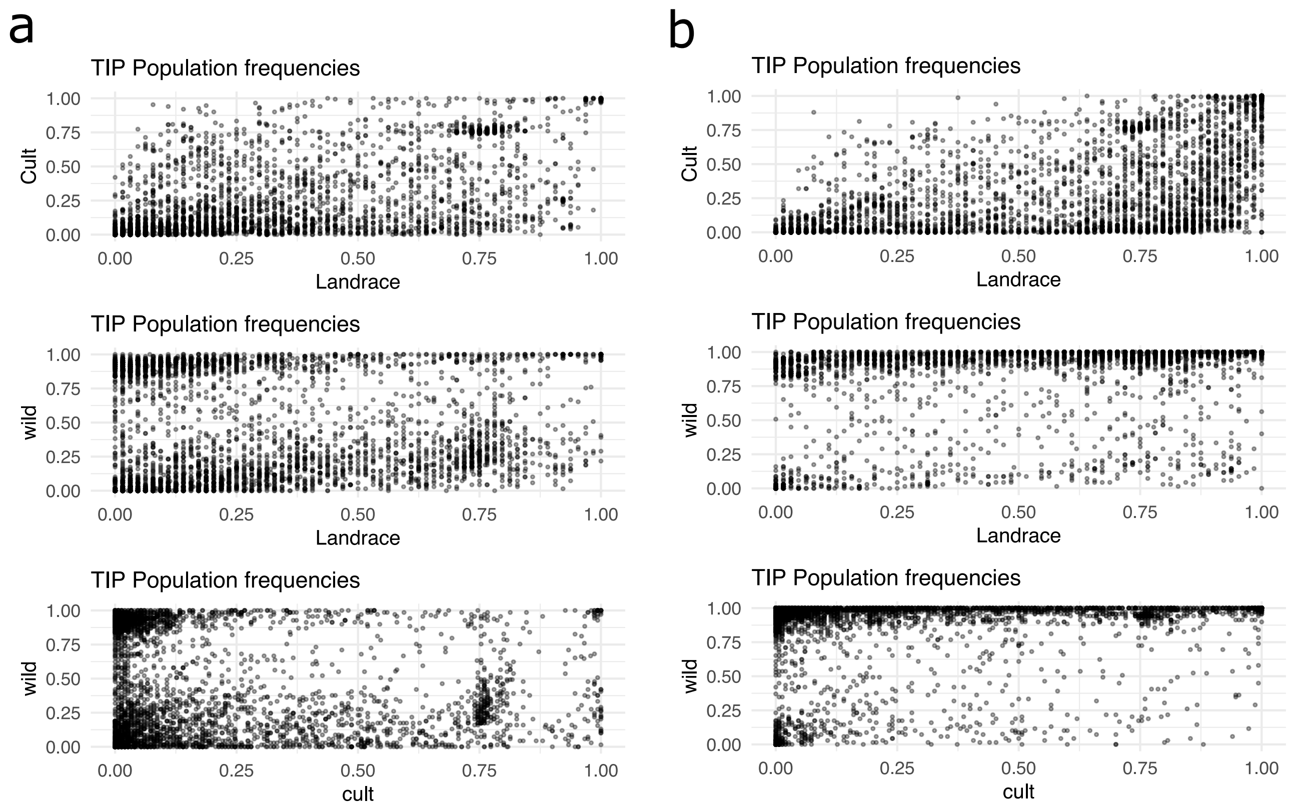

Figure S6. TIP population frequencies in *G. hirsutum*. a) Scatterplots representing the frequency of TIP insertion variants in the three *G. hirsutum* populations. b) Scatterplots representing the frequency of TIP deletion variants in the three *G. hirsutum* populations. Cult = Cultivated accessions.

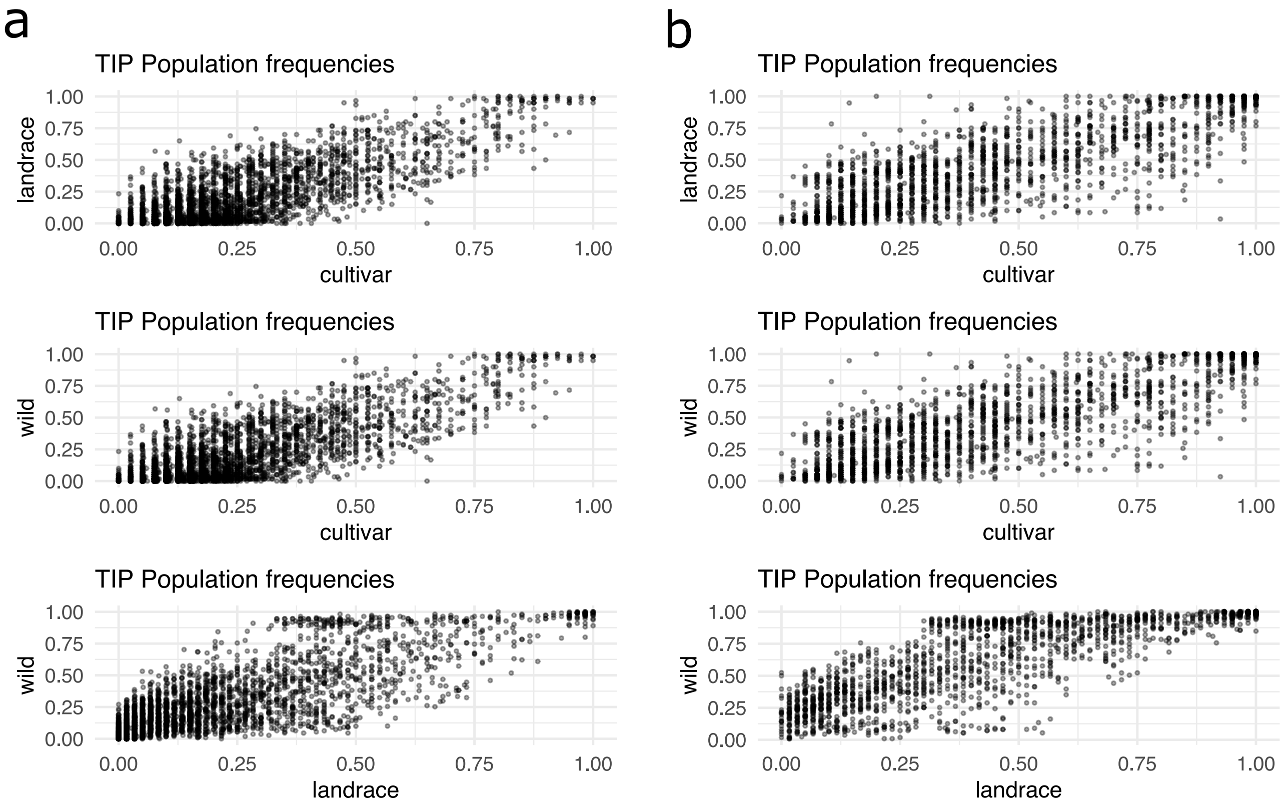

Figure S7. TIP population frequencies in *G. barbadense*. a) Scatterplots representing the frequency of TIP insertion variants in the three *G. barbadense* populations. b) Scatterplots representing the frequency of TIP deletion variants in the three *G. barbadense* populations. Cult = Cultivated accessions.

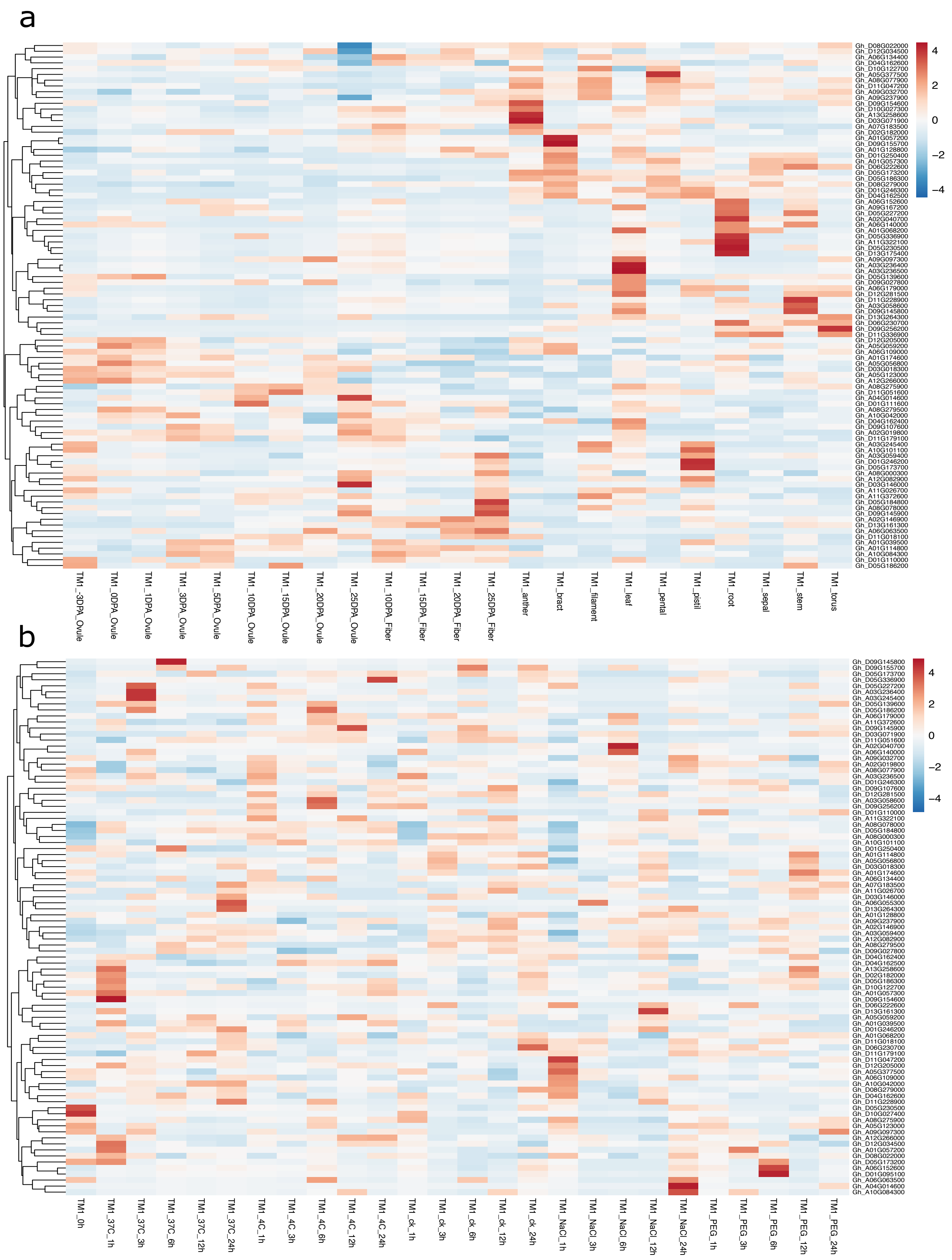

Figure S8. Expression heatmaps of genes near high PBS TIPs in *G. hirsutum*. a) Heatmap representing the relative expression (Rlog values of raw counts) of the genes close to (max 2kbp) high PBS TIPs in different tissues and developmental stages of *G. hirsutum.* Each value is the average of three replicates per sample. b) Heatmap representing the relative expression (Rlog values of raw counts) of the genes close to (max 2kbp) high PBS TIPs in different stress conditions for *G. hirsutum*. Each value is the average of three replicates per sample.
